# Characterization and diversity of the complete set of GH family 3 enzymes from *Rhodothermus marinus* DSM 4253

**DOI:** 10.1038/s41598-020-58015-5

**Published:** 2020-01-28

**Authors:** Kazi Zubaida Gulshan Ara, Anna Månberger, Marek Gabriško, Javier A. Linares-Pastén, Andrius Jasilionis, Ólafur H. Friðjónsson, Guðmundur Ó. Hreggviðsson, Štefan Janeček, Eva Nordberg Karlsson

**Affiliations:** 1 Division of Biotechnology, Dept. of Chemistry, Lund University, P.O. Box 124, SE-221 00 Lund, Sweden; 2 Laboratory of Protein Evolution, Institute of Molecular Biology, Slovak Academy of Sciences, Dúbravská cesta 21, SK-84551 Bratislava, Slovakia; 3 Matís, Vínlandsleið 12, IS-113 Reykjavík, Iceland; 4 Faculty of Life and Environmental Sciences, University of Iceland, Askja, IS-101 Reykjavík, Iceland; 5 Department of Biology, Faculty of Natural Sciences, University of SS Cyril and Methodius, Nám. J. Herdu 2, SK-91701 Trnava, Slovakia

**Keywords:** Biochemistry, Molecular modelling

## Abstract

The genome of *Rhodothermus marinus* DSM 4253 encodes six glycoside hydrolases (GH) classified under GH family 3 (GH3): *Rm*Bgl3A, *Rm*Bgl3B, *Rm*Bgl3C, *Rm*Xyl3A, *Rm*Xyl3B and *Rm*Nag3. The biochemical function, modelled 3D-structure, gene cluster and evolutionary relationships of each of these enzymes were studied. The six enzymes were clustered into three major evolutionary lineages of GH3: β-*N*-acetyl-glucosaminidases, β-1,4-glucosidases/β-xylosidases and macrolide β-glucosidases. The *Rm*Nag3 with additional β-lactamase domain clustered with the deepest rooted GH3-lineage of β-*N*-acetyl-glucosaminidases and was active on acetyl-chitooligosaccharides. *Rm*Bgl3B displayed β-1,4-glucosidase activity and was the only representative of the lineage clustered with macrolide β-glucosidases from Actinomycetes. The β-xylosidases, *Rm*Xyl3A and *Rm*Xyl3B, and the β-glucosidases *Rm*Bgl3A and *Rm*Bgl3C clustered within the major β-glucosidases/β-xylosidases evolutionary lineage. *Rm*Xyl3A and *Rm*Xyl3B showed β-xylosidase activity with different specificities for *para*-nitrophenyl (*p*NP)-linked substrates and xylooligosaccharides. *Rm*Bgl3A displayed β-1,4-glucosidase/β-xylosidase activity while *Rm*Bgl3C was active on *p*NP-β-Glc and β-1,3-1,4-linked glucosyl disaccharides. Putative polysaccharide utilization gene clusters were also investigated for both *R. marinus* DSM 4253 and DSM 4252^T^ (homolog strain). The analysis showed that in the homolog strain DSM 4252^T^ *Rmar_1080* (*Rm*Xyl3A) and *Rmar_1081* (*Rm*Xyl3B) are parts of a putative polysaccharide utilization locus (PUL) for xylan utilization.

## Introduction

Marine extremophilic biotopes, such as hot springs and hydrothermal vents, harbour diverse microbes hitherto underexploited and unexplored. Recent genomic studies show that many of the species, especially those found in coastal geothermal areas surrounded by profusion of carbohydrate rich biomass (seaweeds as well as terrestrial species), contain a wide array of novel glycoside hydrolases (GHs)^1,2^. Thermostable GHs have numerous applications in different fields, making marine thermophiles targets for prospecting of industrially interesting enzymes^3,4^. *Rhodothermus marinus* are Gram-negative marine thermophilic bacteria, previously classified under the phylum Bacteroidetes, but recently assigned to the new phylum Rhodothermaeota^5^. The type-species was isolated from a coastal hot spring on the North-West coast of Iceland and has an optimum temperature of 65 °C and is slightly halophilic^6^. *R. marinus* can utilize a variety of sugars as carbon sources and produces a wide range of GHs^6–12^. Sequence analysis shows that the *R. marinus* genome contains a large number of genes encoding GH enzymes, many of which are secreted extracellularly; yet, several of them appear to be attached to the cell surface^13^. These putative enzymes include six members of GH family 3 (GH3) (*Rm*Bgl3A, *Rm*Bgl3B, *Rm*Bgl3C, *Rm*Xyl3A, *Rm*Xyl3B and *Rm*Nag3), which have not yet been studied.

Glycoside hydrolase family 3 (GH3) is intriguing due to possible roles in cellulosic biomass degradation, bacterial and plant cell wall remodelling, recycling and in pathogen defence^14–17^. GH3 members are retaining enzymes, capable of hydrolysing the terminal glycosidic bond in the non-reducing end of a number of glycosides and glyco-conjugates^18^. According to the Carbohydrate-Active enZYme (CAZy) database^19^ (http://www.cazy.org), this family is widely distributed in bacteria, fungi and plants. It contains more than 27,000 genes (December 2019) encoding putative enzymes and most are of bacterial origin. Members of the family are known for diverse activities, such as β-d-glucosidases, β-d-xylosidases, α-l-arabinofuranosidases and β-*N*-acetyl-d-glucosaminidases. This activity spectrum makes the family interesting for biotechnological applications such as degradation of renewable resources for biofuel production^20,21^. As sequenced-based families group together GHs of different specificity^22^, without biochemical investigation it is difficult to resolve substrate specificities for individual enzymes. Looking at the CAZy database the total number of biochemically characterised enzyme is significantly less compare to total gene numbers^23^.

In this study, we report biochemical characterisation of the complete set of GH3 enzymes from *R. marinus* DSM 4253. The hydrolysis data show that the six enzymes encoded in the genome have non-redundant substrate specificities which will help in future characterisation of other enzymes from this family. This study gave us significant insights into novel structural features of the enzymes as well as on corresponding loci in the *R. marinus* genome.

## Results

### Gene identification and cluster analysis

**Figure 1:**
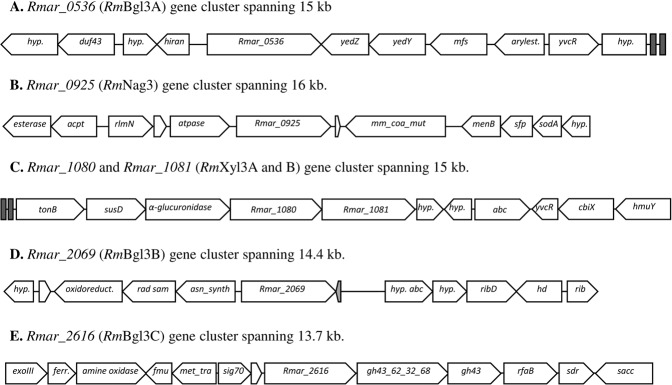
Gene cluster analysis in *Rhodothermus marinus* DSM 4253. The gene cluster corresponding to GH3 loci in strains DSM 4252^T^ and DSM 4253 is identical. (**A**) Downstream of *Rmar_0536* are genes enconding: membrane proteins (*yedZ* and *yedY*); a membrane transporter protein (*mfs*); an arylesterase precursor (*arylest*.); an ABC transporter ATP-binding protein (*yvcR*); a hypothetical protein (*hyp*.). Upstream of *Rmar_0536* are genes encoding: a DNA-binding protein (*hiran*); two hypothetical proteins (*hyp*.); a potential methyltransferase (*duf43*). (**B**) Downstream of *Rmar_0925* are genes encoding: a methylmalonyl-CoA mutase (*mm_coa_mut*); a 1,4-dihydroxy-2-naphthoyl-CoA synthase (*menB*); a 4′-phosphopantetheinyl transferase (*sfp*); a superoxide dismutase (*sodA*); a hypothetical protein (*hyp*.). Upstream of *Rmar_0925* are genes encoding: ATPase (*atpase*); a 23 S rRNA methyltransferase (*rlmN*); an *O*-acetylhomoserine aminocarboxypropyltransferase (*acpt*); a homoserine *O*-acetyltransferase (*esterase*). (**C**) Downstream of *Rmar_1080* and *Rmar_1081* are genes encoding: two hypothetical proteins (*hyp*.); a periplasmic ABC transporter substrate-binding protein (*abc*), a potential cobalamin binding protein (*yvcR*.); a cobalamin biosynthesis protein (*cbiX*); a heme-binding protein (*hmuY*). Upstream of *Rmar_1080* and *Rmar_1081* genes encoding: an α-Glucuronidase (*α-glucuronidase*) and a pair of *susC* (a TonB-dependent receptor) and *susD* homologes. (**D**) Downstream of *Rmar_2069* are genes encoding: a tRNA-Gln-CTG,tRNA (gray arrow); a hypothetical protein similar to ABC transporter (*hyp. abc*); a hypothetical protein (*hyp*.); a riboflavin synthase (*ribD*); a phosphohydrolase (*hd*); a riboflavin. Upstream of *Rmar_2069* are genes encoding: an asparagine synthase (*asn_synth*); a Fe-S oxidoreductase (*rad sam*); a FAD-dependent oxidoreductase (*oxidoreduct*.); a hypothetical protein (*hyp*.). (**E**) Downstream of *Rmar_2616* are genes encoding: a glycoside hydrolase of the GH43_62_32_68 superfamily (*gh43_62_32_68*); a predicted glycosyl hydrolase of the GH43/DUF377 family (*gh43*); a protein similar to glycosyltransferase involved in cell wall biosynthesis (*rfaB*); a short-chain dehydrogenase (*sdr*); a saccharopine dehydrogenas (*sacc*). Upstream of *Rmar_2616* are genes encoding: a RNA polymerase ECF-type sigma factor gene (*sig70*); a tRNA (cytosine-5-)-methyltransferase (*met_tra*); a Fmu domain protein (*fmu*); a flavin containing amine oxidoreductase (*amine oxidase*); a bacterioferritin (*ferr*.); an exodeoxyribonuclease III (*exoIII*).

Six genes encoding glycoside hydrolase (GH) family 3 (GH3) enzymes were identified in the *Rhodothermus marinus* DSM 4253 genome by BLAST analysis, and designated according to the locus tags of their homologs in *Rhodothermus marinus* DSM 4252^T^ (Supplementary Table 2). The positions were in accordance with those of the type strain, and as indicated by the gene number, the four genes *Rmar_0536* (encoding *Rm*Bgl3A)*, Rmar_0925* (encoding *Rm*Nag3), *Rmar_2069* (encoding *Rm*Bgl3B) and *Rmar_2616* (encoding *Rm*Bgl3C) were found at different positions the genome, while *Rmar_1080* (encoding *Rm*Xyl3A) and *Rmar_1081* (encoding *Rm*Xyl3B) were located adjacent to each other (Fig. 1). Clustering with other GH-encoding, regulatory or transporter genes, was investigated by analysing neighbouring genes in both strains.

For *Rm*Bgl3A*, Rm*Nag3 and *Rm*Bgl3B no adjacent glycoside hydrolase encoding genes were found (Fig. 1A,B,D). *Rm*Nag3 is predicted to be co-transcribed with four intracellular proteins including a 23 s rRNA methyltransferase, a putative ATPase and a transcriptional regulator (Fig. 1B). A gene encoding an ABC transporter was located in the vicinity of *Rm*Bgl3B that may be of importance for substrate uptake (Fig. 1D). Signal peptides were predicted for *Rm*Nag3, *Rm*Bgl3B and *Rm*Bgl3C but not for *Rm*Bgl3A.

*Rm*Xyl3A and *Rm*Xyl3B were located in a putative polysaccharide utilization locus (PUL)^24,25^ for xylan utilization (Fig. 1B). This putative xylan utilization locus is also present in the type strain, consisting of six GHs, including the previously cloned and characterized GH10 endo-xylanase *Rm*Xyn10A^26–29^ (encoded by *Rmar_1069*) isolated from the type strain. Table 1 describes the cluster which consists of *Rmar_1067*, annotated as a transcriptional regulator of the LacI family, and the 14 following genes (*Rmar_1067 – Rmar_1081*). Several versions and settings of SignalP were used to predict presence of signal peptides. No signal peptide was found for *Rmar_1075, Rmar_1076* and *Rmar_1081* (encoding *Rm*Xyl3B) which were concluded to code for intracellular proteins, while *Rmar_1071, Rmar_1073* and *Rmar_1080* (encoding *Rm*Xyl3A) code for proteins containing a signal peptide. The other genes predicted signal peptide patterns with lower score. A putative por-secretion system C-terminal sorting domain (CDD: cl22550 in the Conserved Domain Database (CDD)) was encoded in *Rmar_1068, Rmar_1069, Rmar_1071* and *Rmar_1073*. This domain-type has previously been described for *Rm*Xyn10A^13^. Promotors were predicted upstream *Rmar_1067, Rmar_1068, Rmar_1069*, *Rmar_1074* and *Rmar_1075* and termination sites were predicted downstream *Rmar_1067, Rmar_1071, Rmar_1078* and *Rmar_1081*. The cluster includes a TonB-dependent receptor (encoded by *Rmar_1070*) predicted to be co-transcribed with *Rm*Xyn10A, an RNA polymerase σ-factor, the anti-FecI σ-factor FecR, and a pair of *susC* (a TonB-dependent receptor) and *susD* homologs (*Rmar_1075 – Rmar_1078*).

**Table 1:** Putative locus for degradation and uptake of xylan in *Rhodothermus marinus* DSM 4252^T^.

Gene loci	Annotation	Potential cellular location^a^	Potential co-transcription^b^
*Rmar_1067*	LacI family transcriptional regulator	IC	1
*Rmar_1068*	GH43, CBM6	EC and CA	2
*Rmar_1069*	CBM4, CBM4, GH10-*Rm*Xyn10A	EC and CA	2
*Rmar_1070*	TonB-dependent receptor	OM	2
*Rmar_1071*	Hypothetical protein	EC and CA	2
*Rmar_1072*	Hypothetical protein	PS or EC	3
*Rmar_1073*	Hypothetical protein	EC and CA	3
*Rmar_1074*	GH10	PS or EC	4
*Rmar_1075*	RNA polymerase σ^70/24^-factor	IC	4
*Rmar_1076*	anti-FecI σ-factor, FecR	IM	4
*Rmar_1077*	TonB-dependent receptor, SusC/RagA domain protein	OM	4
*Rmar_1078*	SusD/RagB domain protein	EC and CA	4
*Rmar_1079*	α-glucuronidase, GH67	PS or EC	5
*Rmar_1080*	GH3 - *Rm*Xyl3A	PS or EC	5
*Rmar_1081*	GH3 - *Rm*Xyl3B	IC	5

^a^EC - extracellular, CA - cell attached, OM - outer membrane, PS - periplasmic space, IC – intracellular, IM – inner membrane. The cellular location is predicted based on prediction of signal peptides and putative por secretion system C-terminal sorting domain.

^b^Co-transcription is based on predicted operons, Rho-independent translational termination sites and promotors.

A second smaller potential carbohydrate utilization cluster is seen around *Rm*Bgl3C (a β-glucosidase). *Rm*Bgl3C is predicted to be co-transcribed with a cluster of genes. Three genes, two encoding GHs, and a homologue to the RfaB glycosyltransferase (a family GT4 glycosyl transferase) are located downstream *Rm*Bgl3C (Fig. 1E). The two GHs, of which one contains two domains, are classified into GH43 by CDD, but into GH130 by the CAZy database. A signal peptide was predicted for *Rm*Bgl3C, while surrounding genes were predicted to be intracellular (lacking signal peptides).

### Enzyme purification

The activity of all six GH3 candidates was confirmed using the respective cell extract after expression in *E. coli*. Purification by nickel affinity chromatography resulted in enzyme purities of 70–80% for all six enzymes (as judged by SDS-PAGE, Supplementary Fig. 1), which also showed that the molecular mass of each enzyme was in accordance with the theoretical molecular mass. Dependent on the domain composition (see below), the molecular mass of the GH3 enzymes ranged from 66 to 107 kDa (Supplementary Table 2).

### Activity screening

Specific activity was first determined using α- and β-*p*NP-linked sugars at 60 °C (Supplementary Table 3), a temperature where all enzymes were shown to be stable. The results showed that *Rm*Nag3 was only active on *p*NP-β-Glc*N*Ac, which makes it a putative β-*N*-acetyl-d-glucosaminidases. *Rm*Bgl3A showed highest hydrolytic activity towards *p*NP-β-Glc and *p*NP-β-cellobioside (*p*NP-β-Cel) with some activity on *p*NP-β-Xyl. *Rm*Bgl3B was most active on *p*NP-β-Glc, but with significant activity on *p*NP-β-Cel and *p*NP-β-Xyl, and minor activity on *p*NP-α-l-Ara. In contrast, *Rm*Bgl3C only hydrolysed *p*NP-β-Glc (with low specific activity). Both *Rm*Xyl3A and *Rm*Xyl3B showed highest specific activity towards *p*NP-β-Xyl, but displayed side activity on *p*NP-α-l-Ara and *p*NP-β-Glc.

### Temperature and pH optima

The effect of temperature and pH on activity was studied using the *p*NP-substrate with highest specific activity for the respective enzyme (Supplementary Table 4). Highest optimal temperature was observed for *Rm*Nag3 and *Rm*Bgl3B at 90 °C, while the remaining enzymes displayed activity optima between 60–80 °C. The pH optima for all six enzymes were between pH 5.0–5.6 except *Rm*Bgl3C, which was pH 7.0.

### Kinetic analysis

Kinetic parameters on the same substrates (Table 2), showed that *Rm*Bgl3A had a relatively low K_M_ and higher catalytic efficiency (k_cat_/K_M_) for *p*NP-β-Glc compared with *p*NP-β-Xyl (Table 2). *Rm*Bgl3A also had the highest catalytic efficiency for *p*NP-β-Glc of the three enzymes with β-glucosidase activity (*Rm*Bgl3A, B and C). The K_M_ of *Rm*Bgl3C was an order of magnitude higher than the K_M_ of the two other enzymes, showing lower affinity for this substrate. The turnover number for *Rm*Bgl3B (k_cat_) was in the same range for *p*NP-β-Glc, *p*NP-β-Xyl and *p*NP-α-l-Ara. The higher k_cat_/K_M_ of 511 s^−1^mM^−1^ for *p*NP-β-Glc (due to lower K_M_) suggests that this is the most preferred synthetic substrate. All three enzymes, *Rm*Bgl3A, *Rm*Bgl3B and *Rm*Bgl3C, showed substrate inhibition while hydrolysing *p*NP-β-Glc, *Rm*Xyl3A and *Rm*Xyl3B were active against *p*NP-β-Glc, *p*NP-β-Xyl and *p*NP-α-l-Ara, and comparison of kinetic parameters revealed a definite preference for *p*NP-β-Xyl with a low K_M_. *Rm*Xyl3A and *Rm*Xyl3B showed a similar substrate preference, but *Rm*Xyl3B had higher turnover number and catalytic efficiency.

**Table 2:** Kinetic parameters on aryl substrates. Data presented as means ± standard error from three independent experiments.

Enzyme	Substrate	K_m_ (mM)	k_cat_ (s^−1^)	k_cat_/K_M_ (s^−1^ mM^−1^)
*Rm*Bgl3A	*p*NP-β-Glc^a^	0.1 ± 0.0	79.9 ± 3.4	754.9 ± 28.1
	*p*NP-β-Xyl	11.8 ± 1.0	140.8 ± 0.6	12.0 ± 0.0
*Rm*Bgl3B	*p*NP-β-Glc^a^	0.1 ± 0.0	49.5 ± 4.0	511.4 ± 66.1
	*p*NP-β-Xyl	1.2 ± 0.1	65.8 ± 0.4	56.2 ± 0.7
	*p*NP-α-l-Ara	1.9 ± 0.1	47.7 ± 0.8	25.0 ± 1.2
*Rm*Bgl3C	*p*NP-β-Glc	1.6 ± 0.9	67.2 ± 0.5	42.9 ± 2.2
*Rm*Xyl3A	*p*NP-β-Xyl	0.4 ± 0.0	50.1 ± 1.9	140.17 ± 15.7
	*p*NP-β-Glc	6.5 ± 0.8	29.2 ± 0.5	4.5 ± 0.3
	*p*NP-α-l-Ara	8.8 ± 1.0	94.2 ± 11.2	10.7 ± 1.0
*Rm*Xyl3B	*p*NP-β-Xyl	0.3 ± 0.0	160.1 ± 0.6	481.9 ± 21.5
	*p*NP-β-Glc	1.6 ± 0.4	57.5 ± 0.6	36.1 ± 0.9
	*p*NP-α-l-Ara	2.0 ± 0.2	556.8 ± 8.6	280.9 ± 2.4
*Rm*Nag3	*p*NP-β-Glc*N*Ac	0.1 ± 0.0	102.3 ± 3.0	1314.4 ± 50.3

^a^Substrate inhibition was observed for *p*NP-β-Glc at concentration above 2 mM.

The kinetic parameters for *Rm*Ng3 were determined using *p*NP-β-GlcNAc and the K_M_ value was 0.1 mM (Table 2), in a reaction run in the presence of 20 mM sodium phosphate buffer. Additional kinetic analysis was then made using different concentrations of sodium phosphate in 50 mM HEPES buffer (Table 3), to investigate any potential phosphorolytic function for *Rm*Nag3. The K_M_ value increased between 0 and 50 mM of phosphate added to HEPES buffer from 0.16 to 0.30 mM, with no further increase at higher phosphate concentration (100 and 200 mM). For k_cat_ a similar trend was observed (Table 3). However, there was no clear trend in k_cat_/K_M_ values to suggest any significant effect of phosphate on enzyme activity (Table 3).

**Table 3:** Kinetic parameters for aryl substrate hydrolysis by *Rm*Nag3.

Phosphate (mM)	Substrate	K_M_ (mM)	k_cat_ (s^−1^)	k_cat_/K_M_ (s^−1^ mM^−1^)
0	*p*NP-β-Glc*N*Ac	0.2 ± 0.1	144 ± 4	920 ± 50
50	*p*NP-β-Glc*N*Ac	0.3 ± 0.1	223 ± 4	740 ± 20
100	*p*NP-β-Glc*N*Ac	0.3 ± 0.1	189 ± 3	630 ± 10
200	*p*NP-β-Glc*N*Ac	0.3 ± 0.1	232 ± 1	860 ± 30

All the reactions were performed in 50 mM HEPES buffer pH 6.0 in absence or presence of sodium phosphate. Data presented as means ± standard error from three independent experiments.

### Activity on natural substrates

Hydrolysis of natural substrates was monitored using a set of oligosaccharides (Table 4). *Rm*Nag3 was able to release glucosamine from chitobiose and chitopentaose, consistent with the *p*NP-β-Glc*N*Ac hydrolysis, confirming it as a β-*N*-acetyl-glucosaminidase.

**Table 4:** Hydrolysis of oligosaccharides.

Substrate	Specific activity (µmol min^−1^ mg^−1^)					
	*Rm*Bgl3A	*Rm*Bgl3B	*Rm*Bgl3C	*Rm*Xyl3A	*Rm*Xyl3B	*Rm*Nag3
Cellobiose (β-1,4)	333 ± 1	312 ± 0	301 ± 0	6 ± 2	10 ± 2	−^a^
Xylobiose (β-1,4)	67 ± 0	—	—	94 ± 1	244 ± 1	—
Laminaribiose (β-1,3)	—	—	552 ± 0	—	—	—
Cellohexaose (β-1,4)	197 ± 0	—	142 ± 1	22 ± 0	86 ± 1	—
Xylohexaose (β-1,4)	—	—	—	4 ± 0	98 ± 1	—
Xylan (β-1,4)	—	—	—	—	50 ± 1	—
Chitobiose (β-1,4)	—	—	—	—	—	132 ± 1
Chitopentose (β-1,4)	—	—	—	—	—	35 ± 0

Type of linkage for each oligosaccharide is indicated in parentheses and activity were measured based on release of monosaccharides.

^a^No activity was detected.

*Rm*Bgl3A produced glucose from both cellobiose and cellohexaose, which confirmed exo-glucanase activity. In addition, the enzyme maintained its bifunctional activity by hydrolysing xylobiose to xylose. *Rm*Bgl3B hydrolysed cellobiose, but was not active on cellohexaose, limiting the activity to short substrates. *Rm*Bgl3C hydrolysed all glucooligosaccharides tested, but showed clear preference for laminaribiose over cellobiose, and thus a clear preference for hydrolysis of β-1,3- over β-1,4-linkages.

Both *Rm*Xyl3A and *Rm*Xyl3B were hydrolysing xylobiose, but only *Rm*Xyl3B showed exo-glycanase activity and released xylose from both xylohexaose and xylan.

### Phylogenetic analysis of the *R. marinus* GH3 enzymes

**Figure 2:**
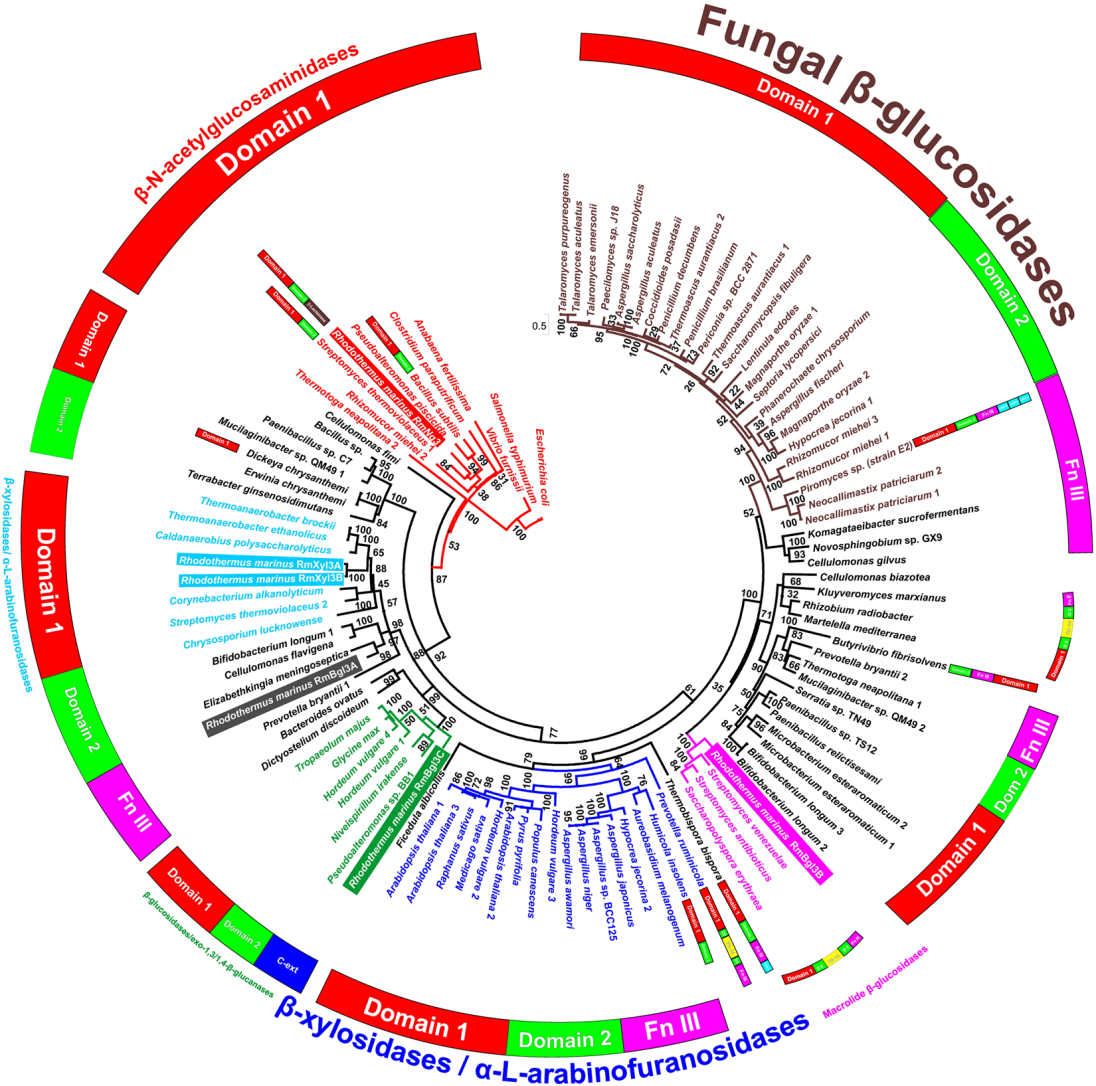
Phylogenetic relationship between proteins in GH3. The maximum likelihood phylogenetic tree was calculated using amino acid sequences of biochemically characterized proteins from GH3 together with six protein sequences from *Rhodothermus marinus* (highlighted in colored boxes). Sequences belonging to particular group are colored in the same color and the schematic representation of domain organization is shown in a circle above the tree. If domain organization of particular protein differs from prevailing domain organization of the group to which it belongs, its domain organization is shown separately next to the protein.

A phylogenetic analysis was performed on the amino acid sequences of 100 characterized GH3 proteins, including the six enzymes from *R. marinus*. Multiple sequence alignment revealed strictly conserved residues within the family and highlights the lack of the otherwise conserved catalytic acid/base among the β-*N*-acetyl-glucosaminidases (Supplementary Fig. 2). Based on the maximum likelihood tree (Fig. 2), it can be seen that the β-*N*-acetyl-glucosaminidase cluster is the deepest rooted group within GH3, separated from others. Characterized enzymes clustering in this lineage have two different domain architectures, single-domain or two-domain enzymes. One subgroup consists of both domain 1 and 2, the other one is missing the C-terminal domain 2. *Rm*Nag3 clusters with the lineage of β-*N*-acetyl-glucosaminidases, but instead of the typical single or two-domain architecture, *Rm*Nag3 displays three domains, containing an additional β-lactamase domain at its C-terminus. BLAST search using the BALSTp tool^30^, revealed a number of gene sequences encoding proteins with similar domain architecture (i.e. domain 1, domain 2 and the β-lactamase domain) e.g. genes encoding putative GH3 candidates from *Salinibacter altiplanensis* (RefSeq: WP_103019701) and *Rhodohalobacter halophilus* (RefSeq: WP_083750206), indicating a common role. To date, *Rm*Nag3 is however the only characterized enzyme with this type of domain architecture.

The other five GH3 enzymes cluster with different subgroups in two main evolutionary lineages. The first lineage consists of the three subgroups: thermostable multifunctional β-xylosidases, closely related β-glucosidases and exo-β-1,3-1,4-glucanases from plants and bacteria (Fig. 2). Four of the *R. marinus* GH3 enzymes cluster with subgroups within this first lineage (*Rm*Bgl3A, *Rm*Bgl3C, *Rm*Xyl3A and *Rm*Xyl3B). *Rm*Bgl3A is part of a weakly defined cluster formed by three bacterial β-glucosidases (Fig. 2), all clustering within the β-glucosidase subgroup. The enzyme consists of domains 1, 2 and the fibronectin type III (FnIII) domain. The protein *Rm*Bgl3C clusters together with exo-β-1,3-1,4-glucanase from plants and bacteria and is a two-domain enzyme consisting of domain 1 and 2. At its C-terminus it has a C-terminal extension, like exo-glucanase (ExoI) from *Hordeum vulgare* (UniP: Q9XEI3), instead of the domain 3 found in e.g. exo-glucanase (ExoP) from *Pseudoalteromonas* sp. BB1 (UniP: Q0QJA3). *Rm*Xyl3A and *Rm*Xyl3B both consist of domain 1, 2 and the FnIII domain and cluster together with the group of thermostable enzymes with predominant β-xylosidase (EC 3.2.1.37) activity with minor α-l-arabinofuranosidase (EC 3.2.1.55) and β-glucosidase (EC 3.2.1.21) activities (Fig. 2). This group is separated from, and therefore not directly phylogenetically related to, other β-xylosidase clusters originating mainly from plants and fungi.

**Figure 3:**
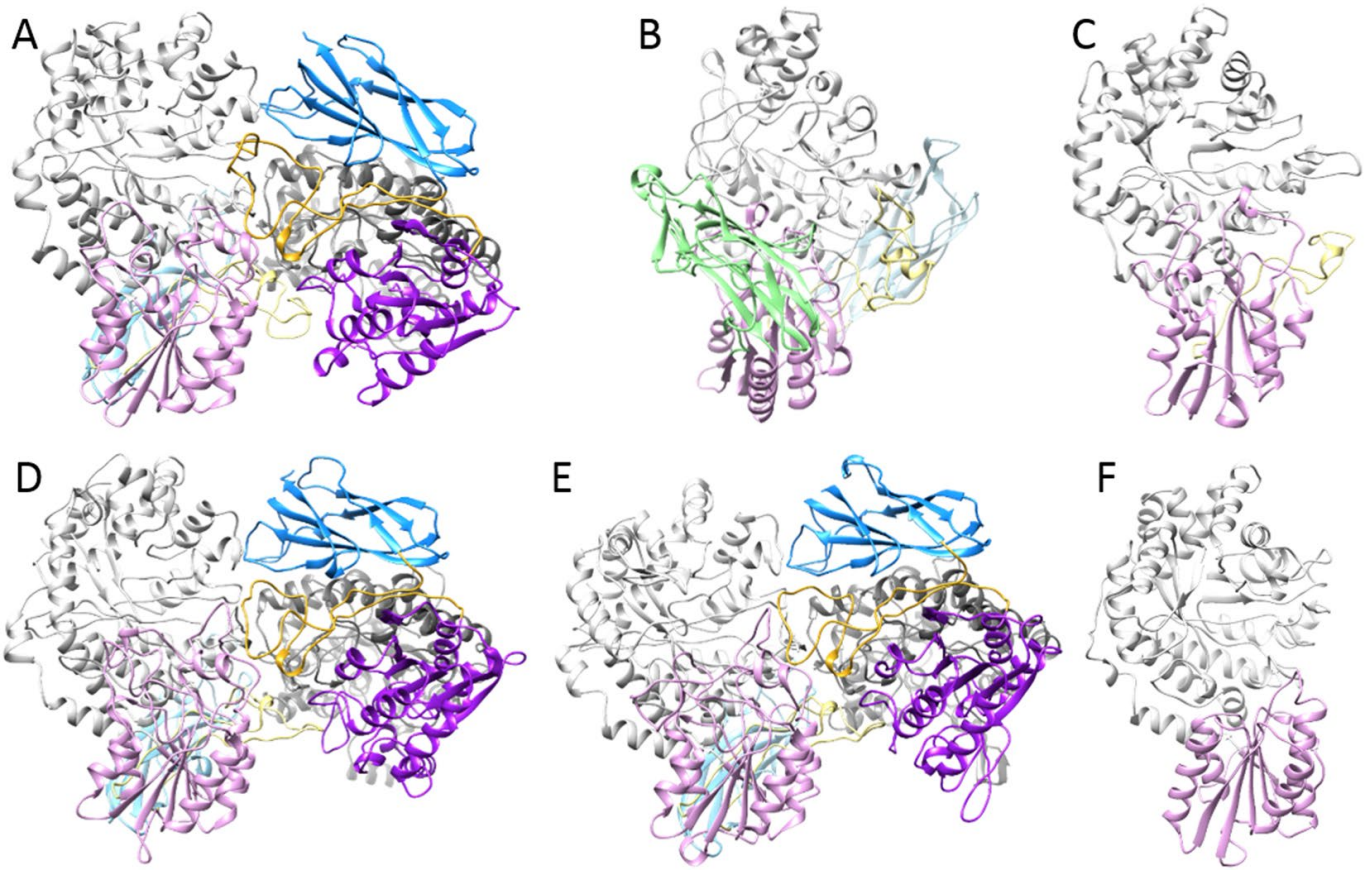
Homology models of the six GH3 enzymes from *Rhodothermus marinus* DSM 4253. Ribbon representation of (**A**) *Rm*Bgl3A, (**B**) *Rm*Bgl3B, (**C**) *Rm*Bgl3C, (**D**) *Rm*Xyl3A, (**E**) *Rm*Xyl3B and (**F**) *Rm*Nag3. Domain 1 is colored in gray, domain 2 in purple, FnIII in blue, PA14 in green and the linker between domain 2 and FnIII in yellow. *Rm*Bgl3A, *Rm*Bgl3B and *Rm*Bgl3C were modelled as dimers, one chain in each dimer is colored in light colors while the other is colored in dark colors. The β-lactamase domain of *Rm*Nag3 was not modelled and is not represented in the figure.

A second lineage is formed by two large subgroups. The first subgroup comprises fungal and bacterial β-glucosidases, including a group of macrolide β-glucosidases, whereas the second subgroup is formed by β-xylosidases from plants, fungi and bacteria (Fig. 2). *Rm*Bgl3B clusters with the macrolide β-glucosidase subgroup together with bacterial (mostly actinobacterial) β-glucosidases involved in activation of secreted antibiotics, activated by the removal of glucosyl moieties from their non-active glycosylated forms. In addition to domains 1, 2 and FnIII, it comprises the PA14 domain (Fig. 3F) inserted between β-strand **k** and α-helix **K**.

### Structural analysis of the *R. marinus* GH3 enzymes

Domain organization, tertiary structure and active site arrangement for the six GH3 enzymes were investigated by homology modelling and compared to closely related structures. Sequence similarities with template structures and model validation are presented in Table 5. Homology model validation show an overall good quality of the six models however indicating some parts of low quality. The modelled structure of *Rm*Bgl3B have low quality in parts of the PA14 and FnIII domains and in loop **l** in domain 2 close to the active site. For *Rm*Xyl3A and *Rm*Xyl3B, several long loops in the modelled structures were not present in the templates, making loop predictions in these regions poor. The *Rm*Nag3 model had very good quality, despite lower sequence identity with its template.

**Table 5:** Validation of the homology models of the six GH3 enzymes from *Rhodothermus marinus*.

Enzymes	Main template			Verify 3D	PROCHECK	ProSA		ERRAT	
	PDB-entry	Resolution (Å)	Sequence identity (%)	Average 3D-1D score ≥0.2^a^ (%)	Ramachandran plot^b^	Z-score		Overall quality factor^c^ (%)	
*Rm*Bgl3A	3U4A	2.20	47	94.9	91.9;7.6;0.2;0.2	−11.3	−10.9	93.8	93.9
*Rm*Bgl3B	4I3G	1.40	44	94.7	89.5;9.9;0.4;0.1	−11.6		92.0	
*Rm*Bgl3C	3WLH	1.65	48	95.0	90.6;8.6;0.4;0.4	−11.4		98.4	
*Rm*Xyl3A	3U48	2.20	34	88.3	90.0;8.8;0.9;0.4	−11.0	−10.4	96.3	94.3
*Rm*Xyl3B	3U48	2.20	36	93.0	89.2;9.9;0.5;0.4	−11.8	−11.1	96.0	94.3
*RmNag3*	3NVD	1.84	30	93.4	92.8;6.8;0.2;0.2	−10.4		97.2	

*Rm*Bgl3A, *Rm*Xyl3A and *Rm*Xyl3B were modelled as dimers, result for chain A and B are showed separately for ProSA and ERRAT analysis.

^a^80% of the residues in a structure is required at an averaged 3D-1D score of 0.2 to be considered a good model.

^b^Numbers corresponds to percentage of residues in: Most favorable regions; additionally allowed regions; generously allowed regions; disallowed regions.

^c^For high-resolution X-ray structures an overall quality factor of 95% is considered a good protein structure and above 91% is expected for X-ray structures with a resolution between 2.5 and 3 Å.

The overall structures of the most common domains in GH3 enzymes: (α/β)_8_ barrel of domain 1, (α/β)_6_ sandwich of domain 2, and β-sandwich of FnIII were conserved, when present in the modelled structures (Fig. 3)^31^. The FnIII domain is found in *Rm*Bgl3A, *Rm*Bgl3B, *Rm*Xyl3A and *Rm*Xyl3B, and is situated on the side of the domain 1 barrel, in contact with domain 2. The PA14 domain of *Rm*Bgl3B is situated on top of the barrel in contact with domain 2. *Rm*Bgl3A, *Rm*Xyl3A and *Rm*Xyl3B were modelled as dimers, with the monomers positioned with the surface created by the FnIII domain, domain 2 and domain 1 next to the opposite chain, and with the linker between domain 2 and FnIII positioned on top of the barrel of the opposite chain.

The active sites are situated on top of the barrel (Supplementary Figs. 3 and 4), are pocket shaped and are made up of both domain 1 and 2, the exception being *Rm*Nag3, which will be discussed separately below. *Rm*Bgl3B has a very deep and narrow active site pocket, and the PA14 domain as well as a long loop **g** covers the surface over the active site. The PA14 domain of *Rm*Bgl3B differs considerably from the PA14 domain present in closely related GH3 enzyme *Km*BglI from *Kluyveromyces marxianus* which have been shown to be involved in substrate interaction^32^. For *Rm*Bgl3A, the linker between domain 2 and the FnIII domain on the opposite chain is involved in shaping the active site. This linker makes the active site deeper on one side, compared to the active site of *Rm*Bgl3C and the β-xylosidases. However, *Rm*Bgl3A displays a shorter loop **b** which opens up the active site on the opposite side. *Rm*Bgl3C has a different conformation of loop **j** on domain 2, making the active site narrower in this direction.

The homology model of *Rm*Nag3, the β-*N*-acetyl-glucosaminidase, displays a well-conserved tertiary structure as well as active site arrangement (Fig. 3F, Supplementary Figs. 3F, 4.F and Table 6). The enzyme consists of domain 1 and 2 but only domain 1 builds up the active site (Supplementary Fig. 3F). Conserved residues important for catalysis were found in positions corresponding to those in structure determined GH3 β*-N*-acetyl-glucosaminidases: catalytic nucleophile Asp118 on β-strand **g**^33^, the Asp227-His229 dyad on loop **e**^34^, and Arg186 and Phe188 on loop **d**^35,36^ (Supplementary Fig. 4F). Similar to other GH3 enzymes, Lys216 and His217 on β-strand **e** and Asp118 on β-strand **c** was found hydrogen bonding to Glc*N*Ac in subsite −1. Superimposition with NagZ from *Pseudomonas aeruginosa* in complex with Glc*N*Ac and l-Ala-1,6-anhydroMur*N*Ac (PDB: 5G3R), showed that Arg60 on β-strand **a**, Arg126 on loop **c** and Glu306 on loop **g** were potentially hydrogen bonding to a sugar unit in subsite +1 (Supplementary Figs. 3F and 4F). All residues important for substrate binding were conserved in the closely related NagA from *Streptomyces thermoviolaceus* (Table 6).

**Table 6:** Comparison of potential substrate interacting residues with closely related characterized GH3 enzymes based on sequence alignment and structure.

Enzyme	Subsite−1				Subsite +1					
*Rm*Bgl3A	D110	G218^*a^	A428*		Y287	Y614	G431	−^b^	R606	F254
*Elizabethkingia meningoseptica* (46%) AAB66561.1	D71	G179	S387		Y248	E587	S390	—	R572	F215
*Rm*Bgl3B	D71	R178	S380		W246	Y703	V383	—	S682*	Y213
*Streptomyces venezuelae* – DesR (44%) PDB: 4I3G	D98	R206	S410		W274	Y691	V413*	—	D670*	Y241
*Rm*Bgl3C	D121	G234*	T450*		Y311	—	W453	—	—	Y278
*Pseudoalteromonas* sp. BB1 – ExoP (45%) PDB: 3F93	D136	G252	S460		W321	—	W463	—	—	F288
*Hordeum vulgare* – ExoI (48%) PDB: 1EX1	D95	G217	T431		W286	—	W434	—	—	Y253
*Rm*Xyl3A	E123	G237*	S446*		W307	—	P449*	Y545	E648*	Y274
*Rm*Xyl3B	E125	G239*	S448*		W309	—	P451*	W547	E650*	Y276
*Caldanaerobius polysaccharolyticus* – Xyl3A (42, 43%) AFM44649.1	E108	G218	A429		Y288	W631	C432	C524	G627	Y255
Enzyme	Subsite−1				Subsite +1					
*Rm*Nag3	D118	R186	D227	H229	R60	R126	R306			
*Streptomyces thermoviolaceus* – NagA (39%) BAA32403.1	D164	R232	D274	H276	R80	R172	G352			

The identities between the enzymes are indicated in parenthesis. Strictly conserved resides presented in Supplementary Fig. 2 are excluded from the table. The table is broken before the β-*N*-acetyl-glucosidases and the columns are not comparable above and under this line.

^**a**^Gap in the sequence alignment.

^b^The residue is not in proximity for substrate interaction based on structures.

**Figure 4:**
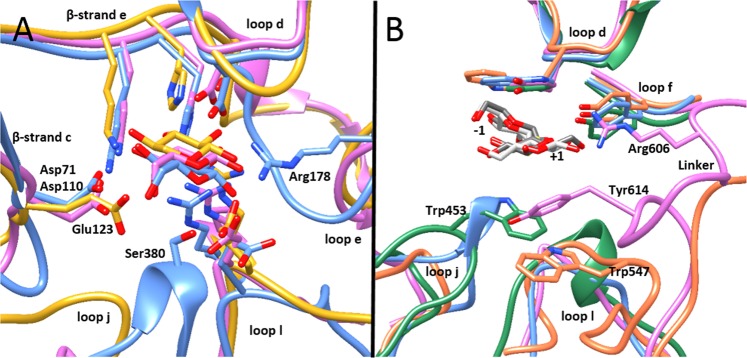
Comparison of active sites of the β-glucosidase and β-xylosidases showing residues involved in (**A**) subsite −1 and (**B**) subsite +1. Superimposition of homology models of *Rm*Bgl3A (pink), *Rm*Bgl3B (blue), *Rm*Bgl3C, *Rm*Xyl3A (yellow) and *Rm*Xyl3B (orange). Cellobiose (gark gray) and laminaribiose (light gray) in subsite −1 and +1 from PDB entries 1IEX and 1J8V respectively, both solved in complex with ExoI from barley. *Rm*Xyl3A and *Rm*Xyl3B have similar −1 and +1 subsites and *Rm*Bgl3C have a similar −1 subsite as *Rm*Bgl3A in terms of possible interacting residues.

The modelled structures of the β-glucosidases and β-xylosidases presented −1 subsite-architectures typical of GH3, with small but important differences (Fig. 4A, Supplementary Figs. 3, 4 and Table 6). The strictly conserved catalytic nucleophile on β-strand **g**^37^, the catalytic acid/base on loop **l** in domain 2^38^, a tandem Lys and His on β-strand **e**, Arg on loop **d** and Met on β-strand **f** were in positions corresponding to those in other GH3-structures^39^. Subsite −1 of *Rm*Xyl3A and *Rm*Xyl3B were very similar. This was also the case for subsite −1 of *Rm*Bgl3A and *Rm*Bgl3C, while *Rm*Bgl3B revealed two non-conserved residues: Arg178 on loop **e** and Ser380 on loop **j** potentially hydrogen bonding with the glucose in subsite −1 (Fig. 4A). A difference between the β-xylosidases and β-glucosidases was found on β-strand **c**; the two β-xylosidases possess a Glu and the three β-glucosidases an Asp, potentially hydrogen bonding with the respective sugar in subsite −1 (Fig. 4A). These features were conserved in the closest relative of the respective enzyme (Table 6).

Subsite +1 was studied by superimposition of structure determined enzyme-ligand complexes (PDB: 1IEX, 1J8V, 4ZO9 and 5AE6) (Fig. 4B, Supplementary Figs. 3, 4 and Table 6). Both β-glucosidases and β-xylosidases displayed an aromatic residue on top of subsite +1 on loop **d**. In *Rm*Bgl3A, *Rm*Bgl3C and the two β-xylosidases an aromatic residue below subsite +1, was potentially sandwiching the sugar unit. This residue was positioned in different loops in the respective enzyme (Fig. 4B). In *Rm*Bgl3A, Tyr614 is located in the linker region between domain 2 and FnIII of the opposite chain. In *Rm*Bgl3C, Trp453 is located in loop **j** in domain 2. In *Rm*Xyl3A and *Rm*Xyl3B, Tyr545 and Trp547, respectively, are located two positions after the acid/base catalyst on loop **l** in domain 2. Due to low sequence identity to the model templates, the prediction of loop **l** in domain 2 is, however uncertain, including the potential involvement of Tyr545 and Trp547 in subsite +1. In *Rm*Bgl3A, an additional residue, Arg606 in the linker between domain 2 and FnIII, is potentially interacting with subsite +1. In *Rm*Bgl3B, *Rm*Bgl3C, *Rm*Xyl3A and *Rm*Xyl3B, a Tyr on loop **f** creates hydrogen bonding possibilities. *Rm*Bgl3A has Phe254 in the corresponding position. The interacting residues in subsite +1 were not completely conserved in the closest related structure of the respective enzyme (Table 6). Tyr614 in *Rm*Bgl3A corresponded to Glu587 in the β-glucosidase from *Elizabethkingia meningoseptica* and the aromatic residue on the bottom of subsite +1 in *Rm*Xyl3A and *Rm*Xyl3B was replaced by Cys524 in Xyl3A from *Caldanaerobius polysaccharolyticus*.

## Discussion

*Rhodothermus marinus* has served as a source of various thermostable enzymes. The Carbohydrate Active enZymes (CAZy) database (http://www.cazy.org)^19^ reports 55 glycoside hydrolase (GH) encoding genes in 33 different GH families and two non-classified genes in the type strain (DSM 4252^T^). Six genes encodes GH family 3 (GH3) candidates and five of them are annotated as β-glucosidases in Kyoto Encyclopedia of Genes and Genomes (KEGG). However, the GH3 members of strain DSM 4253 have different substrate specificities, consistent with differences in their modelled structures. It is very important to mention that the GH3 candidates from both strains of *R. marinus* DSM 4252^T^ and 4253 are homologous. The most common domain architecture in GH3 is either a two domain type: domain 1 and 2, with the catalytic nucleophile in domain 1 and acid/base in domain 2^40^ or a three domain type, with an FnIII-domain combined with domain 1 and 2^41^. Besides these domain architectures, there are two clusters of proteins in the phylogenetic tree containing an additional domain type, PA14, inserted between β-strand **k** and α-helix **K**. Since the two clusters are phylogenetically separated (Fig. 2) and differ in both type and orientation of the PA14 domain, it is likely that they are results of two evolutionary independent insertions.

*Rm*Nag3 has a well-conserved −1 subsite and several possible hydrogen bonding residues in subsite +1 (Fig. 4F). Among the interacting residues in subsite +1 Arg126 was found conserved in almost all structure-determined GH3 β-*N*-acetyl-glucosaminidases. *Rm*Nag3 possesses a signal peptide, a β-lactamase domain and showed optimal activity at high temperature. It was earlier proposed that all β-*N*-acetyl-glucosaminidases were phosphorylases^42^. However, this seems less likely as the presence of phosphate in the kinetic reaction showed little effect on hydrolytic activity of *Rm*Nag3. An increase in K_M_ was observed at 50 mM phosphate, but K_M_ remained unaffected with increasing phosphate concentration. A similar trend was also observed for the β-*N*-acetyl-glucosaminidase from *Herbaspirillum seropedicae* which does not have phosphorylase activity^43^. For the GH3 phosphorylase from *Cellulomonas fimi* the presence of phosphate had significant effect on the hydrolytic activity^42^ compared to *Rm*Nag3. It is also interesting to see that *Rm*Nag3 had no activity on *p*NP-β-Glc while both enzymes from *H*. *seropedicae* and *C*. *fimi* were active on this substrate^42,43^. This narrow substrate specificity could be due to the presence of the β-lactamase domain in *Rm*Nag3. Two main biological roles for β*-N*-acetyl-glucosaminidases are degradation of chitin and peptidoglycan turnover. *R. marinus* possesses an extracellular GH18 chitinase^44^ and an intracellular GH20 β-*N*-acetyl-hexosaminidase, suggesting chitin utilization in *R. marinus*. However, the β-lactamase domain in *Rm*Nag3 suggests association with peptidoglycan turnover as the degradation products have been shown to function as inducers for β-lactamase^45^. β*-N*-acetyl-glucosaminidases involved in peptidoglycan turnover are often but not exclusively intracellular in Gram-negative bacteria^46^. Comparison with peptidoglycan turnover in *Escherichia coli* strain K12 shows that the *R. marinus* type strain genome contains orthologous genes for degrading peptidoglycan and release of Glc*N*Ac, including anhMur*N*Ac and tetrapeptide in the periplasmic space (endolytic murein transglycosylase (MltG), membrane-bound lytic murein transglycosylase (MltD), *N*-acetylmuramoyl-l-alanine amidase (AmiA) and NagZ (ortholog to *Rm*Nag3)). No intracellular orthologs to NagZ, the intracellular *N*-acetylmuramoyl-L-alanine amidase (AmpD) or the permease AmpG for transportation of Glc*N*Ac-1,6-anhydro-Mur*N*Ac-peptides across the inner membrane are found in *R. marinus*. The lack of AmpG makes it likely that *Rm*Nag3 is present in the periplasm. This set-up is found in the Gram-positive *Bacillus subtilis* which lacks AmpG and AmpD, and has a β*-N*-acetyl-glucosaminidase (NagZ) and an *N*-acetylmuramoyl-_L_-alanine amidase (AmiE) in the periplasm^47^. In *B. subtilis*, transportation of Glc*N*Ac and anhMur*N*Ac is believed to be done by the phosphotransferase system enzymes MurP (anhMur*N*Ac) and NagE (Glc*N*Ac). No orthologs for these proteins were found in *R. marinus*, however, AnmK (anhydro-*N*-acetylmuramic acid kinase) and NagK (*N*-acetyl-d-glucosamine kinase) were present as intracellular proteins.

Both *Rm*Bgl3A and *Rm*Bgl3B showed specificity for cellobiose but only *Rm*Bgl3A could hydrolyse cellohexaose. *Rm*Bgl3A is expected to be intracellular while *Rm*Bgl3B, which has a higher temperature optimum and a predicted a signal peptide, is likely exported. *Rm*Bgl3A is a dimer with a conserved subsite −1 and a well-defined subsite +1 including two aromatic residues sandwiching the sugar and Arg606 on the linker positioned between domain 2 and the FnIII domain on the opposite chain. These residues are also conserved in the closest related structure-determined enzymes (Supplementary Table 1). The corresponding residue to Arg606 have been hypothesis by others to be responsible for specificity towards β-1,2- and β-1,3-linked glucose and exclusion of activity on β-1,4-linked glucose^48^. This is not the case, either for *Rm*Bgl3A due to lack of activity on laminaribiose (β-1,3), nor the closest structure-determined enzyme, JMB19063 isolated from compost metagenome, which has activity on cellooligosaccharides^49^. *Rm*Bgl3B has a well-defined subsite −1 with two additional interacting residues (Arg178 and Ser380) compared to *Rm*Bgl3A and a weakly interacting subsite +1 with only two residues, one stacking residue and one hydrogen bonding residue, also conserved in the closest related structure-determined enzymes (Supplementary Table 1). Arg178 on loop e in subsite −1 (Fig. 4A) is conserved in several structure-determined GH3 β-glucosidases, especially within the fungal kingdom. Ser380 is situated on a small α-helix on loop j and is found in several other structures, including many fungal β-glucosidases, but is not as common as Arg178. The inability of *Rm*Bgl3B to cleave longer substrate is most likely a result of the very deep active site cleft created by the PA14 domain. The domain is of the same type and is inserted in the same direction as in the main template, DesR from *Streptomyces venezuelae*, which is placed in the same cluster (Fig. 2) and active on glucosylated macrolides^50^. The PA14 domain in other carbohydrate-active proteins, including the closely related *Km*BglI from *Kluyveromyces marxianus*, have been associated with oligosaccharide binding^32,51,52^. However, no substrate interaction possibility for the PA14 domain was found, neither in the model of *Rm*Bgl3B nor in the structure of DesR.

*Rm*Bgl3C has linkage preference towards β-1,3-linked glucose which is found in laminarin, a polysaccharide present in brown algae^53^, in a bacterial polysaccharide called curdlan^54^ and in various mixed-linkage glucans. It clusters under the linage that consists of exo-glucanases from plants and bacteria. Despite C-terminal extension differences, the exo-glucanase ExoP from *Pseudoalteromonas* sp. BB1 and *Rm*Bgl3C display similar activity on laminarioligosaccharides^55^. *Rm*Bgl3C has a clear signal peptide and higher pH optimum than the other GH3 enzymes in *R. marinus*. Therefore, it is likely that the enzyme is exported out of the outer membrane. Corresponding pH optima were also observed in *Rm*Xyn10A and *Rm*Cel12 which are exported^13,56^. *Rm*Bgl3C displays a subsite −1 similar to *Rm*Bgl3A and a subsite +1 with two aromatic resides sandwiching the sugar, the conserved Tyr311 on loop g and Trp453 on loop j, and Tyr278 with a hydrogen bonding possibility. Trp453 is located in a similar position as Tyr614 of *Rm*Bgl3A but the two residues are places on different loops (Fig. 4B). Trp453 is only found in a few structures including the closest structure-determined enzymes (Supplementary Table 1). The two characterised ExoI from barley (*Hordeum vulgare)* and ExoP, are in similarity with *Rm*Bgl3C β-1,3-β-1,4-glucanases. The structural basis for this specificity has been investigated but not clearly understood^57^. Interestingly, loop e which is involved in shaping subsite +1 is longer in *Rm*Bgl3C than in any of the closest structure-determined enzymes. An accurate conformation of this loop can be important in understanding the specificity of *Rm*Bgl3C, as Arg228 on loop **e** in ExoP was found to hydrogen bond to the glucose in subsite +1, when laminaribiose was modelled into the structure^58^.

*Rm*Xyl3A and *Rm*Xyl3B are both bifunctional β-xylosidases/β-1,4-glucosidases but show different activity patterns. They are 93% identical, positioned next to each other on the chromosome, and thus, likely a result of a relatively recent gene duplication. There are two less similar regions identifiable in the sequences. The first region is located in domain 1 in between β-strand g and α-helix H2, whereas the second one is found in domain 2 on an unusually long loop between β-strand k and α-helix K. *Rm*Xyl3B has endo-β-xylanase activity while *Rm*Xyl3A only hydrolysed xylobiose and, interestingly, cellohexaose. Based on kinetic analysis *Rm*Xyl3B also showed specificity for *p*NP-α-l-Ara. A similar type of bifunctional β-xylosidase/β-1,4-glucosidase has been characterised from *Caldanaerobius polysaccharolyticus*^59^, in the same phylogenetic cluster as *Rm*Xyl3A and *Rm*Xyl3B (Fig. 2). Subsite −1 of *Rm*Xyl3A and *Rm*Xyl3B is similar to that of *Rm*Bgl3A and *Rm*Bgl3C except for one residue potentially responsible for the difference in specificity. The two β-xylosidases/β-1,4-glucosidases display a Glu instead of an Asp on β-strand c in subsite −1 (Fig. 4A). This difference could affect the preference for glucose or xylose in subsite −1 as the Asp is making no steric hindrance for the additional CH_2_OH group of glucose. The same pattern was found in other GH3 enzymes (Table 6), including ExoI from barley and Xyl3B from *Prevotella bryantii* where substitution of Glu115 to an Asp increased catalytic activity of Xyl3B on *p*NP- β-Glc^39^. Another interesting feature is the aromatic residue on loop j in domain 2, two positions away from the potential acid/base catalyst in *Rm*Xyl3A and *Rm*Xyl3B (Fig. 4B), with the possibility to be in a position analogous to Tyr614 in *Rm*Bgl3A. This was not found in the *Caldanaerobius polysaccharolyticus* β-xylosidase/β-1,4-glucosidase, but comparison with structures of other GH3 enzymes, reveals an aromatic residue two positions after the acid/base catalyst and a loop j of similar length in several fungal β-glucosidases with very low sequence identity to the *R. marinus* β-xylosidases/β-1,4-glucosidases (see PDB: 4IIB, 5FJI, 5FJJ, 5NBS, 4DOJ and 3ZYZ), and stacking with a glucose in subsite +1 has been shown for AaBGL1 from *Aspergillus aculeatus*^60^.

The genomic context of the GH3 enzymes was informative to some extent concerning their potential role in utilization of carbohydrate substrates. The genome comparison between *R. marinus* DSM 4252^T^ and 4253 showed that all the corresponding GH3 gene loci are identical. In *R. marinus* DSM 4252^T^ and DSM 4253, the putative PUL consists of six GHs: the endo-1,4-β-xylanase *Rm*Xyn10A^13^ with putative location in the extracellular space, an uncharacterized GH10 (a family containing mainly endo-1,4-β-xylanases) and the two GH3 β-xylosidases characterized in the current study, suggesting that the cluster is involved in utilization of 1,4-β-linked xylans. The potential extracellular GH43 belongs to subfamily 15 with no characterized proteins so far. The uncharacterized GH67 is annotated as an α-glucuronidase in NCBI, suggesting that xylan substituted with glucuronic acid or methyl glucuronic acid could be utilised by the PUL. Besides glycan degrading enzymes, a canonical PUL involves a pair of *susC* and *susD* homologues and a regulator^25^, components which are all found in the described cluster. SusC and SusD, first described as vital components of a starch utilization system (SUS) in *Bacteroides thetaiotaomicron*, are co-regulated genes involved in coordinated binding, transport and degradation of carbohydrates from the extracellular space via the outer membrane into the periplasmic space^24^. SusC is a type of TonB-dependent receptor, an outer membrane transporter known to transport ferric chelates, but also shown to transport carbohydrates. TonB-dependent transporters require energy and three inner membrane proteins in a complex, TonB-ExbB-ExbD, for the transportation^61^. SusD is a lipoprotein attached to the outer cell membrane shown to bind carbohydrates. In addition, the putative regulation system identified in the cluster, called trans-envelope signalling, involving an extracytoplasmic function (ECF) σ-factor/anti-σ-factor system is found in PULs from Bacteroidetes^24^. Biochemical and structural characterization of the hydrolytic enzymes involved in the cluster, transcriptome analysis during growth on different substrates as well as knock-out of the genes are necessary to fully understand the mechanisms involved in the PUL described in this study.

In summary, the biochemical and structural characterisation of the GH3 enzymes from *R. marinus* DSM 4253 (and 4252^T^), shows that the six GH3 enzymes encoded in the genome have non-redundant substrate specificities which are involved in extracellular laminarin, potential macrolide degradation, as well as intracellular cellobiose to glucose conversion, the conversion of xylans, and recycling of peptidoglycans, giving significant insights into structural features important for the specificity of these enzymes as well as the organization of corresponding loci in the *R. marinus* genome.

## Materials and Methods

### Chemicals

Laminaribiose, cello-, xylo- and acetyl-chitooligosaccharides were obtained from Megazyme (Wicklow, Ireland). Sodium acetate, laminarin from *Laminaria digitata*, xylan from birch wood, *para*-nitrophenol and all *para*-nitrophenyl-β-d-glycosides were purchased from Sigma-Aldrich (St. Louis, Mo). All other chemicals were of molecular biology or analytical grades and purchsed from VWR International (Stockholm, Sweden).

### Bacterial strains, genome sequencing and gene cluster analysis

*Rhodothermus marinus* strain DSM 4253 was isolated from an intertidal hot spring in Iceland, at a location close to that of the *Rhodothermus marinus* DSM 4252^T^ (Type strain)^6^. The genome was sequenced using TrueSeq chemistry for library construction and MiSeq sequencing platform (unpublished data). Sequencing data was assembled using GS De Novo Assembler software (Roche) and annotated using the RAST annotation server at rast.nmpdr.org^62^. Genes, encoding enzymes of glycoside hydrolase (GH) family 3 (GH3), were identified by BlastX^63^ using GH3 amino acid sequences from the *R. marinus* type strain retrieved from the Carbohydrate-Active enZYme (CAZy) database^19^ (http://www.cazy.org) as query sequences. Sequence regions harbouring the GH3 genes along with flanking sequences, spanning approximately 15 kb, were extracted from the genome sequence using the Geneious molecular biology tool. The structure of corresponding loci of the GH3 genes was resolved in a genome viewer and the neighbouring genes were analysed by BLAST^63^.

Gene clusters were analysed in the genome of the type strain^12^ by annotations presented in the National Center for Biotechnology Information (NCBI) Nucleotide database, Reference Sequence (RefSeq): NC_013501.1, of genes in proximity to the six genes (*Rmar_0536, Rmar_0925, Rmar_1080, Rmar_1081, Rmar_2069*, and *Rmar_2616)* encoding the putative GH3 enzymes: *Rm*Bgl3A, *Rm*Nag3, *Rm*Xyl3A, *Rm*Xyl3B, *Rm*Bgl3B, and *Rm*Bgl3C. Additional annotations were done by compiling the information from searches in the Conserved Domain Database (CDD) from NCBI^64^ and in the CAZy database in the case of annotated GHs. Operons were predicted by DOOR 2.0^65,66^ and Genome2D^67^. Putative promotors were predicted by PePPER^68^ and Rho-independent translational terminator sites were predicted by ARNold^69^ and DOOR 2.0. Potential co-transcription was based on predicted operons, promotors and transcription terminators. Predictions of signal peptides were done by SignalP versions 3.0 and 4.1^70–72^. BlASTp^30^ was used to investigate poorly annotated genes and search for domains. Potential cellular location was based on prediction of signal peptides and the putative por-secretion system C-terminal sorting domain.

### Sequence alignment and evolutionary relationships

From 301 characterized protein members classified in GH3 of the CAZy database, 100 with known activity were selected (Supplementary dataset file). Amino acid sequences of these proteins were retrieved mainly from the UniProt database^73^ and some were obtained from GenBank^74^ and RefSeq^75^ databases. All sequences of characterized GH3 proteins together with those of the six proteins from *R. marinus* were aligned using the programme Clustal-X^76^ and then the alignment was further manually fine-tuned.

Maximum likelihood phylogenetic tree^77^ was calculated using the PhyML algorithm^78^ available through T-REX server (http://www.trex.uqam.ca/^79^. Gamma shape parameter and proportion of invariable sites were estimated by the PhyML itself. Number of relative substitution rate categories was set to four and WAG substitution model^80^ was used. The starting tree was calculated using BIONJ. NNI was used for a tree improvement. Tree topology and branch lengths were optimized. Reliability of tree topologies was evaluated using the bootstrap test^81^ with 100 replications. For the phylogenetic analysis, only amino acid sequences of domain 1, which is universally present in all proteins from GH3, was used.

### Structure homology modelling

Homology modelling of the six GH3 of *R. marinus* DSM 4253 was carried out using the YASARA program^82,83^ with default settings except templates that were manually inserted (Supplementary Table 1). For each enzyme, a BLASTp search^63^ in the Protein Data Bank (PDB) was done and the five enzymes with the highest score were chosen. If several structures were available, the structure without mutations, with a high resolution and relevant ligand was chosen. If the sequence identity was more than 10% units lower than the hit with the highest sequence identity, this template was not used. Only relevant ligands were kept in the structure and in some cases ligands were manually modified: in *Rm*Xyl3A and *Rm*Xyl3B into a xylose and in the case of *Rm*Nag3 into a β-*N*-acetyl-glucosamine before entering the homology modelling. Only ions conserved among the templates were kept in the structures. Only dimerization conserved among the templates were kept and in other cases only chain A was kept. The β-lactamase domain of *Rm*Nag3 was removed before homology modelling since no hit was found for the entire protein and no hit with a sequence identity above 30% was found for the domain alone. YASARA homology modelling generates five alignments for each template and builds a model for each alignment. A hybrid model is generated by combining the best part of the models. Each hybrid model was manually checked by superimposition and comparison of templates and other GH3 structures in Chimera^84^. Side chains in the active site and ligand positioning were manually changed based on conserved features in the closest related structures. The peptide bonds linking the conserved residues Lys and His on β-strand **e** and the following two amino acids were manually modified into cis-conformation, which are conserved within GH3. Modified models were energy minimised and then refined in YASARA. Refinement was done with default setting, only changing temperature and pH to the optima for each enzyme (Supplementary Table 4), with a 500 ps simulation of molecular dynamics with the YASARA2 force field^85^. During the simulation, 20 trajectories were saved, energy minimised and analysed by checking the energy of the system as well as dihedral angles, packaging1D and packaging3D. The trajectory with best overall quality was further evaluated. Evaluation of each refined structure was done manually and by several online validation tools. Superimposition in Chimera verified that the active site arrangement was kept and stabilized by the simulation. If not, modifications of the hybrid model were revised and a new version was refined. The quality of each refined structure was evaluated by average 3D-1D score generated by Verify3D^86,87^, Ramachandran plot generated by PROCHECK^88^, Z-score generated by ProSA-web^89,90^ and overall quality factor generated by ERRAT^91^. For models containing ions, refinement and evaluation with and without the ions were done. In case of a lower quality with the ions, indicated by ERRAT, the homology modelling and refinement were rerun after deletion of the ion(s) in the templates.

### Cloning and expression of GH3 genes

*R. marinus* DSM 4253 was grown in 500 mL of DIFCO™ Marine Broth (BD, NJ, USA) at 65 °C for 12 hours. The cell pellet was harvested by centrifugation and washed twice with 200 mM sodium phosphate buffer pH 7. Genomic DNA was extracted using ZymoBead™ Genomic DNA Kit (Zymo Research, CA, U.S.A). The GH3 encoding genes, without signal peptide, were amplified using the primers listed (Supplementary Table 2). A C-terminal His-tag was introduced in the primer-designs, except for *Rmar_2069*. Genes digested with *Nde*I and *Bgl*II were ligated into pJOE3075^92^, a non-commercial vector and transformed to *Escherichia coli* BL21 C43. *Rmar_2069* was digested by *Nde*I and *Hind*III, propagated in vector pUC19 followed by sub-cloning into pET-21b(+) (Novagen, Madison, WI) in frame with the C-terminal His-tag and transformed to *E. coli* BL21 C43. Expression was performed at 0.5 L cultivation scale in Erlenmeyer flasks at 37 °C in LB containing 100 µg/mL ampicillin. After reaching an optical density at 620 nm of 0.5, gene expression was induced by 1 mM IPTG for the pET-construct and 0.2% (w/v) L-rhamnose for pJOE3075-constructs^92^. Production was continued at 30 °C over-night. Cells were harvested by centrifugation at 5000 × g for 15 min at 4 °C and washed twice with 20 mM sodium phosphate buffer pH 7.0.

### Purification

Cell pellets were resuspended in binding buffer (20 mM sodium phosphate buffer, 500 mM NaCl, 20 mM imidazole, pH 7.4), and lysed by sonication 5 × 3 min, at 60% amplitude and a cycle of 0.5 using a 14-mm titanium probe (UP400 S; Hielscher Ultrasonic GmbH, Teltow, Germany). For *Rmar_0925* encoding the *Rm*Nag3, the binding buffer contained 20 mM Tris-HCl instead of sodium phosphate buffer Cell debris was removed by centrifugation (14000 × g, 20 min, 4 °C), prior to purification by nickel affinity chromatography using an ÄKTA^TM^ start system (GE Healthcare) with a HisTrap FF crude 1 mL column (GE Healthcare). Bound protein was eluted using gradient of imidazole 20–500 mM and fractions of 1 mL were collected. All purified proteins were stored at 4 °C.

### Hydrolysis of *para*-nitrophenyl glycosides

Enzyme catalysed hydrolysis of *para*-nitrophenyl (*p*NP)-linked substrates was assayed spectrophotometrically at 405 nm using a UV-1650PC spectrophotometer (Shimadzu, Kyoto, Japan) connected to a JulaboMB (Labortechnik GMBH, Germany) temperature-controlled system at 60 °C. Final reaction volume was 600 µL and contained 1 mM substrate dissolved in 20 mM citrate phosphate buffer at pH 5.6. The reaction was initiated by adding 0.04–0.2 µM of enzyme to the pre-incubated reaction solution and monitored for 5 min. 1 U equals the amount of enzyme required to release 1 µmol *p*NP min^−1^. The extinction coefficient for *p*NP at 60 °C is 1426 M^−1^cm^−1^ and 18072 M^−1^cm^−1^ at pH 5.6 and 6.0 respectively. For kinetic parameters, substrate concentrations were 0.05–20 mM, at reaction conditions and enzyme concentration as above except for *Rm*Ng3. Additional the kinetic assays for *Rm*Ng3 were performed at pH 6.0 using 50 mM HEPES buffer, while in the presence of phosphate the buffer was supplemented with 50 mM, 100 mM and 200 mM of sodium phosphate. Each reaction was monitored for 10 min and K_M_, V_max_ and K_i_ values were calculated from GraphPad Prism V6. For *Rm*Nag3, the kinetic parameters for determining phosphorolytic activity were obtained by using KinTek Explorer. The pH and temperature optima were determined in the pH-range 3.0–6.0 (50 mM sodium citrate phosphate buffer), and 7.0–8.0 (50 mM sodium phosphate buffer) and at 40–90 °C in assays with a protein concentration of 0.04–0.1 µM and 1 mM of *p*NP-β-Glc or *p*NP-β-Xyl. All reactions were run in triplicates.

### Hydrolysis of oligosaccharides

Oligosaccharides (10 mg/mL) with a degree of polymerization (DP) of 2 (cellobiose, laminaribiose and xylobiose) and DP 6 (cellohexaose and xylohexaose) in 500 µl, 20 mM citrate phosphate buffer, pH 5.6 were incubated with 5–10 µg/mL enzyme in duplicate reactions in a ThermoMixer (HLC Biotech, Bovenden, Germany) at 60 °C, 600 rpm. Samples (30 µL) were withdrawn at 20 min intervals for 120 min, and diluted with 970 µL 0.5 mM NaOH before analysis on high-performance anion exchange chromatography with pulsed amperometric detection (HPAEC-PAD) (Thermo Fisher Scientific, Waltham, USA), using a CarboPac PA200 column (250 mm × 3 mm, 5.5 µm) and a guard column (50 mm × 3 mm) (Thermo Fisher Scientific, Waltham, USA) of the same material. A mobile phase of 100 mM NaOH at 0.5 mL/min and a linear gradient of sodium acetate (0–120 mM) was used for 25 min. Standards included glucose, cellooligosaccharides (DP 2 to 6), xylose and xylooligosaccharides (DP 2 to 6). An identical method was applied to analyse hydrolysis of acetylated chitooligosaccharides, with *N*-acetylated chitooligosaccharides (DP 2 to 5) and glucosamine as standards.

## Supplementary information

Supplementary Information.

Dataset 1.

Supplementary Information.
