# Supplementary material for: Characterization and diversity of the complete set of GH family 3 enzymes from Rhodothermus marinus DSM 4253
Source: Sci Rep. 2020 Jan 28;10:1329. doi: 10.1038/s41598-020-58015-5 (PMC6987092; doi:10.1038/s41598-020-58015-5)
Supplement: Supplementary file 1 — Supplementary Information. [file 41598_2020_58015_MOESM1_ESM.pdf]

Characterization and diversity of the complete set of GH family 3 enzymes from *Rhodothermus marinus* DSM 4253

Kazi Zubaida Gulshan Ara[1], Anna Månberger[1], Marek Gabriško[2], Javier A. Linares-Pastén[1], Andrius Jasilionis[1], Ólafur H. Friðjónsson[3], Guðmundur Ó. Hreggviðsson[3,4], Štefan Janeček[2,5], Eva Nordberg Karlsson[1]

[1] Lund University, Dept. of Chemistry, Biotechnology, P.O. Box 124, SE-221 00 Lund, Sweden

[2] Laboratory of Protein Evolution, Institute of Molecular Biology, Slovak Academy of Sciences, Dúbravská cesta 21, SK-84551 Bratislava, Slovakia

[3] Matís, Vínlandsleið 12, IS-113 Reykjavík, Iceland

[4] Faculty of Life and Environmental Sciences, University of Iceland, Askja, IS-101 Reykjavík, Iceland

[5] Department of Biology, Faculty of Natural Sciences, University of SS Cyril and Methodius, Nám. J. Herdu 2, SK-91701 Trnava, Slovakia.

Corresponding author:

Kazi Zubaida Gulshan Ara (zubaida.gulshan_kazi@biotek.lu.se)

Eva Nordberg Karlsson (eva.nordberg_karlsson@biotek.lu.se)

Supplementary Figure 1: SDS-PAGE of GH3 enzymes after primary purification using crude FF column. *Lane 1*, molecular mass marker; *lane 2, Rm*Bgl3C; *lane 3, Rm*Bgl3A; *lane 4, Rm*Xyl3A; *lane 5, Rm*Xyl3B; *lane 6, Rm*Bgl3B and *lane 7, Rm*Nag3. Molecular mass marker was run additionally in *lane A1, A2 and A3.*

Supplementary Figure 2. Comparison of conserved regions of GH3. The conserved sequence regions from biochemically characterized GH3 proteins together with six protein sequences from *Rhodothermus marinus* DSM 4253 are shown (color labelled according to the phylogeny cluster). The catalytic residues are highlighted in red and white inversion and substitutions are cyan. The conserved residues are in dark grey and semi-conversed residues are in light grey. Elements of the secondary structure bearing the particular conserved sequence region according to are shown above the alignment.

Supplementary Figure 3. Surface representation of the six GH3 enzymes illustrating how the different domain architecture builds up the active site. (A) *Rm*Bgl3A, (B) *Rm*Bgl3B, (C) *Rm*Bgl3C, (D) *Rm*Xyl3A, (E) *Rm*Xyl3B and (F) *Rm*Nag3. Residues potentially involved in subsite +1 are labeled and loops of interest are marked. All pictures are taken from the same position after superimposition except for *Rm*Bgl3B as the PA14 domain then covers the active site completely. Domain 1 is colored in gray, domain 2 in purple, FnIII in blue, PA14 in green and the linker between domain 2 and FnIII in yellow. One chain in each dimer is colored in light colors while the other is colored in dark colors.

Supplementary Figure 4. Ribbon representation of (A) *Rm*Bgl3A, (B) *Rm*Bgl3B, (C) *Rm*Bgl3C, (D) *Rm*Xyl3A, (E) *Rm*Xyl3B and (F) *Rm*Nag3. Residues important for ligand binding in subsite -1 and possible important in subsite +1 are shown. Some conserved residues marked in Supplementary Fig. 2 are shown but not labelled to make the picture less cluttered. All pictures are taken from the same position (same as in Supplementary Fig. 3 with the exception of *Rm*Bgl3A) after superimposition, the PA14 domain in *Rm*Bgl3B is deleted. Domain 1 is colored in gray, domain 2 in purple, FnIII in blue and the linker between domain 2 and FnIII in yellow. One chain in each dimer is colored in light colors while the other is colored in dark colors.

Supplementary Figure 1.

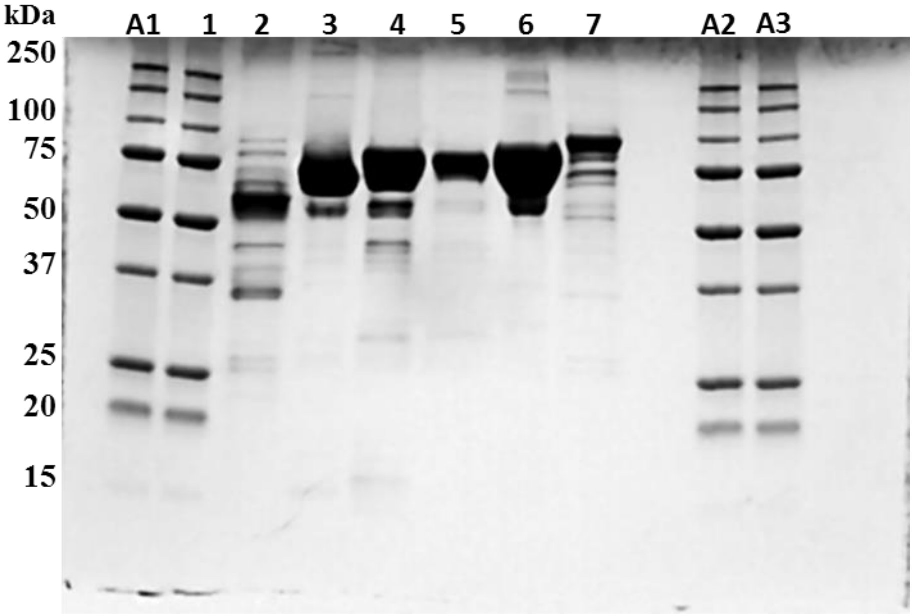

Supplementary Figure 2.

	β-strand c	loop d	β-strand e	β-strand f	β-strand g	loop l
Thabr	43_ALIH-EESCSG	106_PRWGRTEETF	143_VATGKHFVGY	189_SSIMPGYHE	223_IVVSDYFA	468_ESRDR
Thaet	97_ALIH-EESCSG	160_PRWGRTEETF	197_VATGKHFVGY	243_SSIMPGYHE	277_IVVSDYFA	522_ESRDR
Calpo	103_ALVH-EESCSG	166_ARWGRVEETF	203_MATVKHFVGY	249_ASVMAAYHE	283_LVVSDYFG	522_DDCTS
**Rhoma_*Rm*Xyl3A**	118_VIFH-EEALHG	181_PRWGRIEETF	222_IATLKHMAGH	268_LSVMASYNE	302_VIVSDWHG	543_EAYAP
**Rhoma_*Rm*Xyl3B**	120_VLFH-EEALHG	183_PRWGRIEETF	224_IATLKHMTGH	269_LSVMASYNE	304_VIVSDWFA	545_EAWAV
Coral	104_AIVH-EECLTG	167_PRWGRVDECI	200_HATLKHFLGY	246_RSVMNSYTD	280_VVVSDYFS	507_EGNDV
Strth_2	106_AVAH-EECLAG	169_LRWGRVEETI	202_VATLKHFAGY	248_RSVMAAYTE	282_TVVADYFA	509_EGCDV
Chrlu	120_AFVQ-TEGLHG	183_LRHGRVEEMY	216_SAMVKHFAAF	262_YSIMTAYHS	296_FTMTDAGA	522_EHVDV
Biflo_1	90_LIIG-DDCIHG	154_TRWGRVDETF	197_LACAKHFAGY	242_GTFMLGYES	276_TLITDWDN	523_ETCST
Celfl	55_LLIA-EDCIHG	119_LRWGRVSETF	156_LATAKHFAGY	201_ATFMLGYQS	235_TLVTDWDN	497_EGRST
Elime	66_MIFG-MDVIHG	125_PRWGRVSEGS	164_LACVKHFALY	209_GSVMASFNE	243_FIVTDYTG	473_ESSSR
**Rhoma_*Rm*Bgl3A**	105_LLFA-LDVIHG	164_ARWGRIVEGS	203_LATAKHFAAY	248_LSIMSAFNE	282_LVVSDYTS	507_EAASR
Prebr_1	110_ALFH-EEVLSG	173_PSFNRLEESY	210_GACSKHYLGY	248_KALMPGYHA	282_MVVSDYTA	526_ENRDR
Arath_3	100_TYEWWSEALHG	180_PRWGRGQETP	221_AACCKHYTAY	270_ASVMCSYNQ	304_YIVSDCDS	512_ESRDR
Rapsa	95_TYEWWSEALHG	175_PRWGRGQETP	216_AACCKHYTAY	265_ASVMCSYNK	299_YIVSDCDS	507_ESRDR
Arath_1	90_SYKWWSEALHG	170_PRWGRGQETP	211_AACCKHYTAY	260_ASVMCSYNQ	294_YIVSDCDS	502_EGHDR
Medsa	95_KYEWWSEALHG	175_PRWGRGQETP	216_AACCKHYTAY	265_ASVMCSYNK	299_YIVSDCDS	508_ESLDR
Horvu_2	94_AYEWWSEALHG	174_PRWGRGQETP	217_AACCKHYTAY	266_ASVMCSYNK	300_YIVSDCDS	510_ESLDR
Arath_2	90_GYEWWSEALHG	170_PRWGRGQETP	209_AACCKHYTAY	258_ASVMCSYNQ	292_YIVSDCDS	500_ETRDR
Pyrpy	87_GYEWWSEALHG	166_PRWGRGQETP	205_AACCKHYTAY	254_ASVMCSYNQ	288_YIVSDCDS	498_EFRDR
Popca	82_GYEWWSEALHG	162_PRWGRGQETP	200_AACCKHFTAY	249_ASVMCSYNQ	283_YIVSDCDS	482_EGHDR
Horvu_3	85_AYKWWSEALHG	166_PRWGRGQETP	210_SACCKHFTAY	259_SGIMCSYNR	293_YITSDCDA	503_EEVDR
Aspaw	109_AYQVWSEALHG	187_PVWGRGQETP	226_AATAKHYAGY	275_QSVMCAYNA	311_YVSSDCDA	527_EALDR
Aspni	109_AYQVWSEALHG	187_PVWGRGQETP	226_AATAKHYAGY	275_QSVMCAYNA	311_YVSSDCDA	527_EALDR
Aspsp	109_AYQVWSEALHG	187_PVWGRGQETP	226_AATAKHYAGY	275_HSVMCAYNA	311_YVSSDCDA	527_EALDR
Aspja	100_PYQVWSEALHG	178_PVWGRGQETP	218_AATAKHFAGY	267_HSFMCSYNA	303_YVSGDCGA	516_EAQDR
Hypje_2	103_NYQVWNEALHG	182_PLWGRGQETP	222_AATVKHFAGY	271_RSLMCAYNS	307_YVSSDCDA	512_EGADR
Aurme	83_VYQWWQEALHG	165_PRWGRGQETP	204_TATCKHFAGY	253_GAFMCTYSA	290_WVTSDCDS	498_EGMDR
Humin	76_AYNWWSEALHG	155_PRWGRGSETP	192_IATCKHYAGY	241_GSIMCSYNR	278_YITSDCEA	488_EGVDR
Preru	65_KFFWWSEALHG	144_-RWGRGQETY	184_WACAKHYAVH	231_REVMCAYQR	265_LVVSDCGA	625_EGFKG
Fical	106_PYNWNTECLRG	184_-LWGRNQETY	223_SAGCKHFSVH	271_YSFMCSYNR	305_YVVSDEGA	520_EAKDR
Thbbi	82_SFKTGTEALHG	163_-RWGRNEEGY	202_APTLKHYLAN	247_TGVMTAYNL	280_LNVTDAGA	621_EDHD-
Aspac	86_GGMCLQDSPLG	152_PDGGRNWEGF	185_VATAKHYILN	242_GAIMCSYNQ	276_FVMSDWGA	509_EGYIS
Aspsa	86_GGMCLQDSPLG	152_PDGGRNWEGF	185_IATAKHYILN	242_GAVMCSYNQ	276_FVMSDWGA	509_EGYIS
Talem	86_PGLCMQDSPLG	152_PDAGRNWEGF	185_IACAKHFILY	239_GSVMCSYNQ	273_FVMTDWGG	505_EGYIT
Thrma_2	87_RGLCLQDSPLG	153_PDGGRNWEGF	186_IACAKHFIGN	243_GSFMCSYNQ	277_FVMSDWGA	510_EGYIN
Pende	86_WGMCMQDSPLG	152_PDGGRGWEGF	185_IACAKHFIMN	242_GSVMCSYNQ	276_FVMSDWGA	511_EGYIS
Cocpo	86_RSLCMQDGPLG	152_PDGGRNWEGF	185_IACVKHFIVN	242_GSVMCSYNQ	276_FIMSDWQA	511_EGFIS
Talac	82_RSLCMQDSPLG	148_PEGGRNWEGF	181_IACAKHYIGN	235_GSVMCSYNQ	269_FVMTDWSA	503_EGYIN
Talpu	82_RSLCMQDSPLG	148_PEGGRNWEGF	181_IACAKHYIGN	235_GSIMCSYNQ	269_FVMTDWGA	503_EGYIT
Pamsp	84_RGLCLQDGPLG	150_PDGGRNWEGY	183_IATAKHYLLN	237_VSIMCSYNQ	271_FVMSDWGA	504_EGYIS
Persp	95_PGFCTQDSPLG	161_PKGGRNWEGF	194_IACTKHFILN	249_GSIMCSYNK	283_FTMSDWDA	515_EGYIT
Penbr	104_KGFCTQDSPQG	170_PEGGRNWEGF	203_IACAKHYIGN	260_GSFMCSYSQ	294_FVMSDWGA	528_EGYIT
Hypje_1	86_PSLCLQDGPLG	152_PQGGRNWEGF	185_QATAKHYILN	229_ASVMCSYNK	263_YVMTDWNA	472_EGYIT
Magor_2	70_PQLCLQDGPTG	136_PEGGRNWEGF	169_QATAKHYLLN	213_ASVMCSYNK	247_YVMSDWNA	453_EGYLL
Aspfi	71_PSLCLQDSPLG	137_PNGGRNWEGF	170_QACAKHWLGN	214_ASVMCSYNK	248_YIMSDWNA	464_EGYIT
Phach	134_TGLCLQDSPVG	199_PAAGRNWEGG	232_QACAKHFINN	276_ASVMCSYNQ	310_YVMSDWWA	527_EGYLT
Rhimi_1	74_PSLCLEDGPPG	139_PQTGRGWEGF	172_IATAKHYIGN	216_GAIMCSYNQ	250_LIMSDWGA	469_EEYIV
Rhimi_3	81_PSLCLEDSPLG	146_PNAGRAWEAF	179_IATAKHYIGN	223_GSIMCAYNQ	257_FVQSDWGA	476_EEYIT
Sacfi	102_PNLCLQDGPLG	168_ARGGRNFEAF	201_MACVKHFIGN	261_GSVMCSYNR	295_FVVSDWAA	533_EGYLI
Thrma_1	100_PNLCLMDSPLG	166_PRGGRNWEGF	199_IATLKHFIGN	249_GSVMMAYND	283_FVMTDWLT	511_ENYIT
Magor_1	91_PGLCLHDAGNG	157_PVAGRNWEGF	190_IATTKHFIGN	240_ASAMCSYQR	274_FIMLDWNA	503_EGWDR
Seply	88_SGLCLADGPVS	154_PLGGRNWESF	187_QANRKHFIGN	242_ASVMCSYNR	276_YVVSDWYA	524_EGTDR
Lened	103_PEFCLQDGPIG	170_PLNGRGWEGT	203_ATVSKHWIAY	262_NHIMCSYNR	296_GVVSDWGG	529_EGADA
Neopa_1	71_KGLCLQDGPAG	137_PAAGRIWESF	170_IATSKHFVGN	215_NAIMSSYNA	249_MVMSDWWS	455_EEYIV
Neopa_2	69_KGLCLQDGPAG	135_PASGRIWESY	168_IATSKHFVGN	213_NAIMSSYNA	247_MVMSDWWG	453_EEYIT
Pirsp	69_KGLCLQDGPAG	135_PASGRVWENF	168_IVAAKHYVAN	213_GSVMASYNA	247_FVMSDWWA	452_EEYIV
Celgi	105_PALFETDAGQG	175_PRNGRNFEYA	209_ISTLKHFVLN	253_GSVMCAYNR	287_YVMSDWGA	491_EANDA
Novsp	93_PALKETDASLG	163_PRNGRTFEYL	196_ISTIKHFALN	241_GAIMCAYNR	275_FVMSDWGA	483_EGLDQ
Komsu	90_PDLQISDAGLG	161_PRGGRNFEYA	194_ISTLKHYAMN	239_GAVMCSYNR	273_FVMSDWGA	477_EGMDA
Sccer	77_PQIRGTDGPAG	142_PQAGRNFETF	175_MTSAKHYAAN	219_TSVMCAYPK	253_WVMSDWTA	560_ETADR
Stran	34_PEMRATDGPAG	99_PYAGRNFETF	132_IAATKHYAAN	176_GSVMCAYNK	210_WVTSDWLA	522_EDGDR
Strve	92_PELRAADGPNG	158_PHGGRNYETF	191_MTTAKHFAAN	235_ASFMCAYNG	269_WVMSDWLA	578_EGVDR
**Rhoma_*Rm*Bgl3B**	65_PAMRLTDGPAG	130_PEAGRNFETF	163_MATVKHYVAN	207_AAVMCAYNR	241_WVMTDWFA	591_EGRDR
Biflo_2	39_SYMI-TDGPHG	116_PLGGRCFEYW	149_GTSLKHFAAN	194_WTIMCSYNR	228_IVMSDWGA	425_EGFDR
Biflo_3	39_GYMI-TDGPHG	116_PLGGRCFEYW	149_GTSLKHFAAN	194_WTIMCSYNR	228_IVMSDWGA	421_EGFDR
Mices_1	45_SFSM-SDGPHG	122_PRCGRNFEYL	155_GASLKHFAAN	200_WTVMCSYNR	234_AVVSDWGA	425_EGFDR
Mices_2	121_SVFL-TDGPHG	197_PLGGRNFEYF	230_GASLKHFAVN	275_WTVMASYNR	309_LVVSDWGA	498_EGIDR
Paere	41_SVMM-TDGPHG	117_PLCGRNFEYF	150_GTSLKHFAAN	195_WTVMCSYNK	229_FVVSDWGA	368_EGFDR
Paesp_2	31_SIMM-TDGPHG	107_PLCGRNFEYF	140_GTSLKHFAVN	185_WTVMSAYNR	219_IVVSDWGA	411_EGYDR
Sersp	46_NIVM-TDGTYG	144_PLGGRSYEYY	177_GASLKHFACN	222_WTVMSSYNR	256_VVVADWHG	449_EGSDR
Mucsp_2	116_SITM-SDGPAG	190_PLGGRNFEYY	223_DTSIKHYAAN	268_WTVMSSYNK	302_FVMTDWFG	510_EGADR
Thene_1	53_AFVL-ADGPAG	126_PLCGRNFEYY	159_GACIKHFVAN	204_WSVMSAYNK	238_FVMSDWYA	458_EGYDR
Prebr_2	85_QTVV-ADGPAG	158_PLCGRNFEYY	191_GVSAKHFAVN	236_WTLMSSYNR	270_IVMTDWIG	491_EGLDR
Butfi	81_NAQT-CDGPAG	149_PLCGRNFEYY	182_IATPKHFALN	227_GASCLQYNI	261_VVVSDWWG	480_EGWDR
Marme	47_AVKV-SDGPNG	117_GLNGRNFECY	150_GATIKHFVAN	195_WAVMTGYNR	229_IFMSDWFG	568_EGHDL
Rhzra	36_KIKV-TDGPNG	106_GLNGRNFECY	139_AATIKHFVAN	184_KAVMSSYNK	218_VVMSDWFG	559_EGLDL
Kluma	40_AVRV-SDGPNG	109_PLGGRGFESF	142_AATVKHFVCN	187_VCIMTAYNK	221_MLMSDWFG	590_EGYDR
Celbi	43_EVRL-SDGPTG	112_VLGGRLFEAY	145_GACLKHLVAN	190_WSVMAAYND	224_LVMSDWFA	586_ESVDK
Bctov	125_PCIYGVDQIHG	65_PRWARMWENY	106_AACMKHYMGY	151_LSVMVNSGV	185_LIVTDWAD	534_ETPGN
Dicdi	170_PMIYGLDSVHG	235_PLWSRIYETF	279_VCTAKHYFGY	325_GTIMINSGE	359_VAVTDWQD	583_ETPGD
Glyma	112_PMIYGIDAVHG	177_PRWGRCYESY	225_AACAKHYLGD	270_STVMISYSS	304_FVISDWQG	514_ETFGD
Troma	113_PMIYGIDAVHG	178_PRWGRCYESY	226_AACAKHFVGD	271_ATVMVSYSS	305_FVISDWEG	515_EMQGD
Horvu_4	111_PMIYGIDAVHG	176_PRWGRCYESY	223_AACAKHYVGD	268_STVMTSYSS	302_FVISDWQG	512_ETFGD
Nivir	137_PVLFGIDAVHG	202_DRWGRTYEGF	245_VASAKHFLAD	290_LTVMASFSS	324_FIVGDWNA	528_EFQGD
Psesp	130_PTVWGTDAMHG	195_DRWGRTYESY	237_IATAKHFVGD	282_QSVMASFNS	316_FVVSDWNA	520_EWFGD
Horvu_1	115_MIYG-IDAVHG	179_PRWGRCYESY	227_AACAKHFVGD	272_STVMISYSS	306_FVISDWEG	516_ETKGD
**Rhoma_*Rm*Bgl3C**	115_PLLYGIDAVHG	180_IRWGRTYESF	219_LATAKHFAGD	272_GSIMVSYSS	306_IVISDYNA	506_EFLGD
Celfi	84_PLLISANLEGG	149_RNPITNTRTF	184_AASAKHFPGD	236_GHIMLPAYS	279_LVVSDSTT	485_EWAEP
Bacsp	160_ANNS-SDPRHG	232_PRWFRFGMTF	278_NAMVKHWPGG	333_SAVMPYYTI	375_VVCTDWGI	583_EAGGN
Paesp_1	162_VNNS-SDPRHG	234_PRWSRFDGTF	280_NAMVKHWPGG	335_SAIMPYYTI	377_VVCTDWGI	586_EAGGN
Mucsp_1	185_NNNS-SDPRHG	257_PRWARVSGTF	303_NAMVKHWPGG	358_AAVMPYYTI	400_VVCTDWLV	606_KNGGT
Dckch	152_LTIS-TDPRSS	223_PRWPRIDGTF	263_ISIVKHWVGY	312_AGIMPTYSI	356_VILSDWLI	534_EQPHK
Erwch	152_LTIS-TDPRSS	223_PRWPRIDGTF	267_ISIVKHWVGY	316_AGIMPTYSI	356_VILSDWLI	534_EQPHK
Tergi	158_VTVS-TDPRHA	229_PRWARQAGTF	267_ACTSKHFPGG	316_AGIMPYYGM	360_VVVTDWEL	537_ES---
Escco	56_RLVVAVDQEGG	126_ISAAIGERSY	159_KTTGKHFPGH	207_DAIMPAHVI	244_VIFSDDLS	-----
Salty	56_HLVVAVDQEGG	126_ISAAIGERSY	159_KTTGKHFPGH	207_DAIMPAHVI	244_VIFSDDLS	-----
Vibfu	56_PILIGVDQEGG	123_DCRAIGNRAF	156_ATTGKHFPGH	201_DAMMPAHVI	238_IVFSDDLS	-----
**Rhoma_*Rm*Ng3**	112_PLLIAQDTEWG	179_MNPIINVRAF	211_IATAKHFPGH	259_QSIMTGHLA	299_LVVTDALE	492_FVRSG
Bacsu	117_PLMLSIDQEGG	184_DNPVIGVRSF	217_ASALKHFPGH	264_DMVMTAHVQ	314_VIVTDALN	517_VKNDP
Psepi	99_PLFIAIDQEGG	171_NNPVINVRSF	204_LSALKHFPGH	252_GMIMTAHIQ	301_VTVTDALD	489_PPQSA
Strth_1	159_LLIA-TDQEHG	226_ANPVIGVRSF	259_AATAKHFPGH	306_DSIMTAHLQ	345_VVITDSLG	538_VTAG-
Clopa	114_LFIG-FDEEGG	183_PIIGVRSYGS	214_IPTVKHFPGH	261_DMVMTAHIM	299_VIITDDLE	-----
Anafe	76_MFFT-LDHEGG	138_QNPEIGSRAF	171_VGCAKHFPGH	221_QLIMTAHIL	260_VVVSDDLD	-----
Rhimi_2	74_PLFIAVDQENG	142_LNPVIGVRSY	175_ATSIKHFPGH	226_TSVMVGHML	264_VIITDCLE	500_EYTP-
Thene_2	57_RFIVSSDHEGG	123_GSAVVDLRSF	156_IPCIKHFPGH	204_TAVMTAHVK	241_LVLSDAME	433_PFDAK

Supplementary Figure 3.

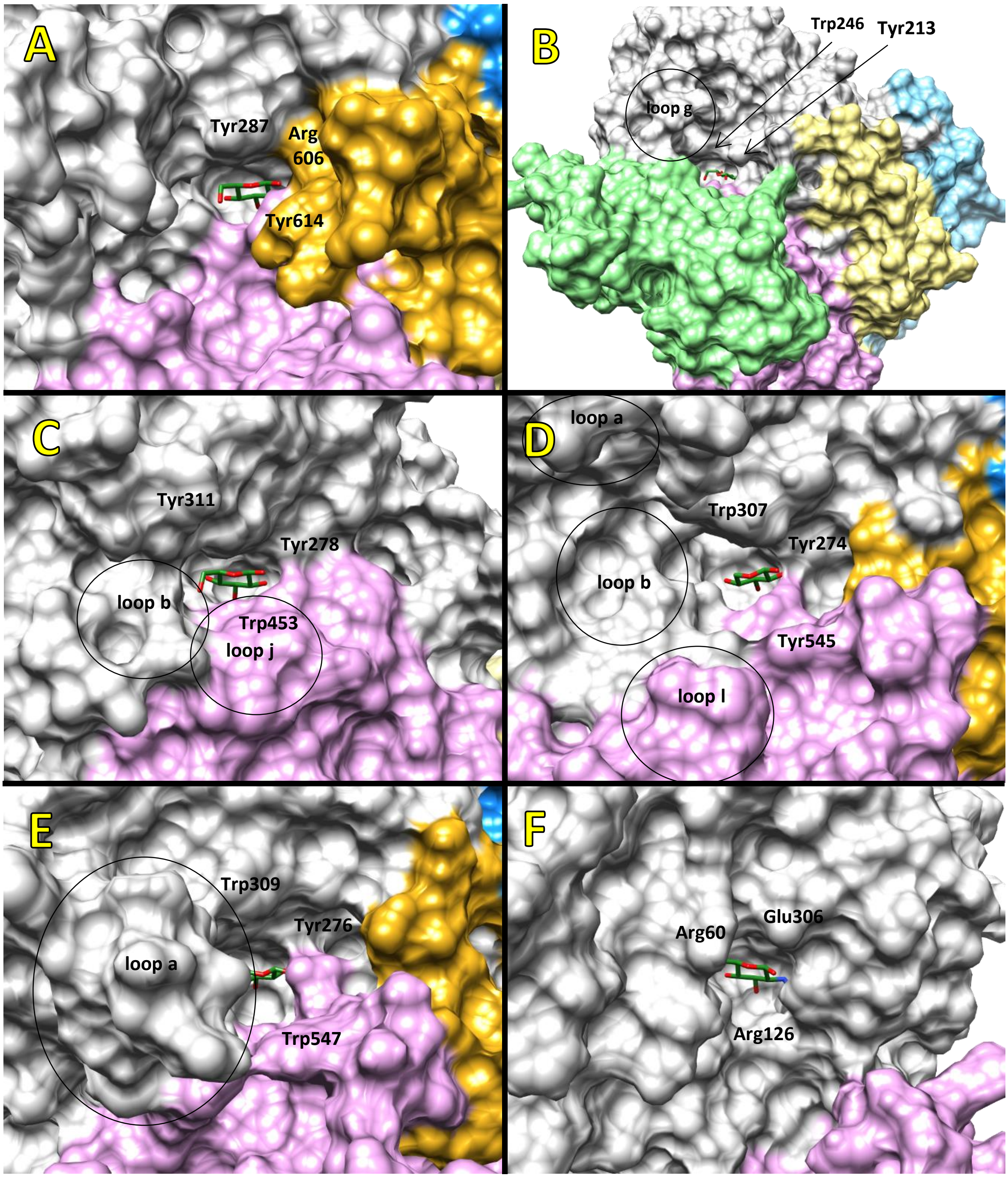

Supplementary Figure 4.

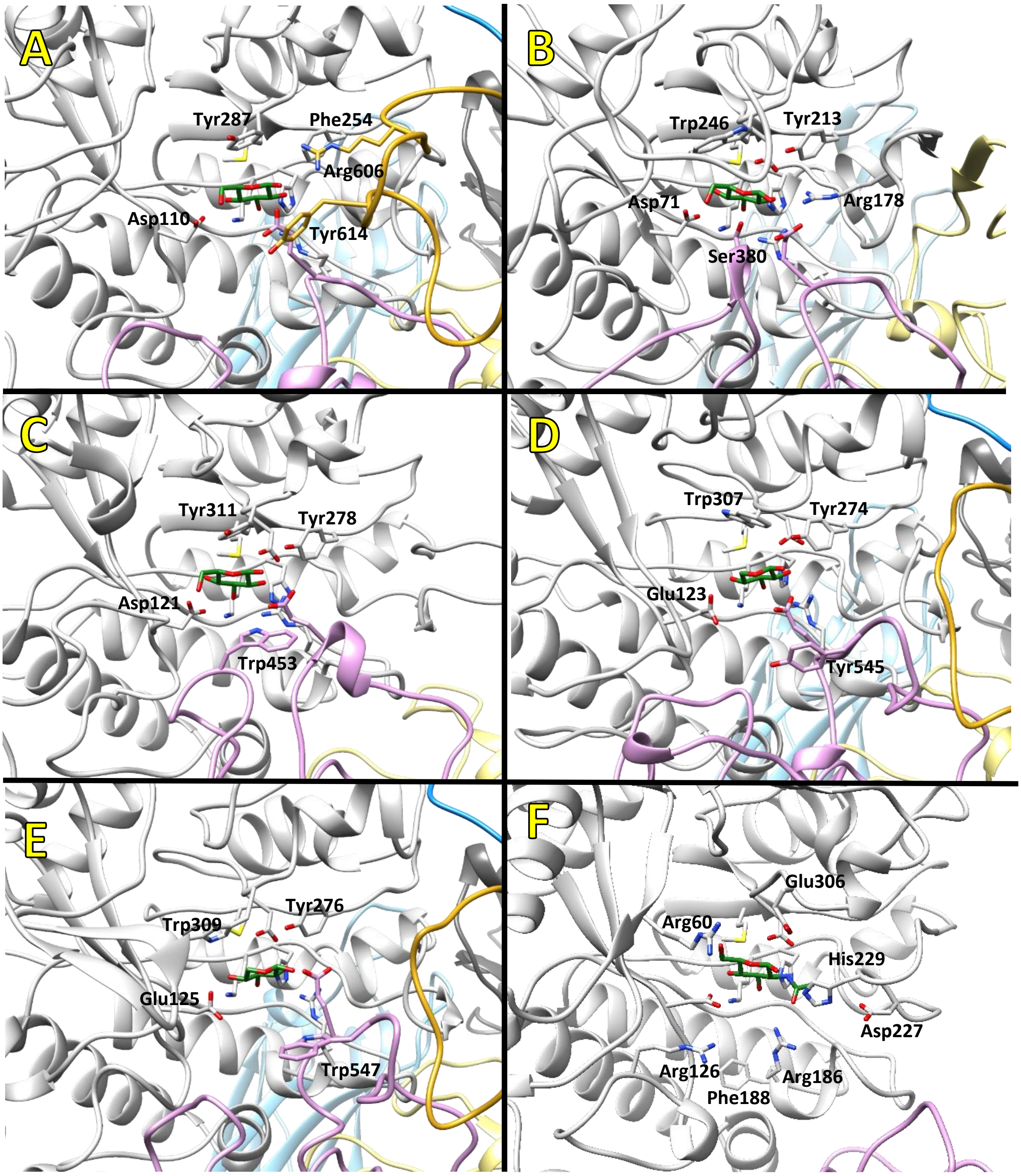

Supplementary Table 1. Templates added into the homology modelling. The enzyme in bold were used as main template.

Enzyme	PDB-entry	Enzyme	Source	Covery (%)	Identity (%)
Rmar_0536 (*Rm*Bgl3A)	**3U4A**	**JMB19063**	**Compost metagenome**	**94**	**47**
	5XXM	BT_3567	*Bacteroides thetaiomicron*	96	46
	4ZOD	Lin1840	*Listeria innocula*	94	41
	5TF0[a]	BACINT_00768	*Bacteroides intestinalis*	94	45
Rmar_0925 (*Rm*Nag3)	**3NVD**	BsNagZ	***Bacillus subtilis***	**84**	**30**
	3SQM	SYNPCC7002_A0075	*Synechococcus* sp.	66	43
	4ZM6	RmNag	*Rhizomucor miehei*	87	31
	5BZA[a]	CbsA	*Thermotoga neapolitana*	52	31
Rmar_1080 (*Rm*Xyl3A)	**3U48**	**JMB19063**	**Compost metagenome**	**95**	**34**
	4ZOD	Lin1840	*Listeria innocula*	94	33
	5Z9S	BlBG3	*Bifidobacterium longum*	95	34
	5TF0	BACINT_00768	*Bacteroides intestinalis*	85	35
	5XXM[a]	BT_3567	*Bacteroides thetaiomicron*	96	32
Rmar_1081 (*Rm*Xyl3B)	**3U48**	**JMB19063**	**Compost metagenome**	**86**	**36**
	5Z9S	BlBG3	*Bifidobacterium longum*	95	35
	4ZOD	Lin1840	*Listeria innocula*	94	33
	5XXM	BT_3567	*Bacteroides thetaiomicron*	96	33
	5TF0	BACINT_00768	*Bacteroides intestinalis*	85	35
Rmar_2069 (*Rm*Bgl3B)	**4I3G**	DesR	***Streptomyces venezuelae***	**97**	**44**
	3ABZ	*Km*BglI	*Kluyveromyces marxianus*	97	32
	5K6M	GlyA1	metagenome	65	43
Rmar_2616 (*Rm*Bgl3C)	**3WLH**	ExoI	***Hordeum vulgare***	**93**	**48**
	5M6G	SACE_6502	*Saccharopolyspora erythraea*	93	55
	3USZ	ExoP	*Pseudoalteromonas* sp. BB1	93	45

[a] Not used in the hybrid model.

Supplementary Table 2. Primers used for cloning GH3 genes from *Rhodothermus marinus* DSM 4253 genome.

Gene loci and Accession No.	Protein	Molecular Weight (kDa)	Sequence orientation	Primers[a]
Rmar_0536	*Rm*Bgl3A	81.3	Forward	5′-CCAATTGCATATGGATCGGGCCCGCGAGGAC
MG457245			Reverse	5′-CGCAGATCTCGGACTGACCACCTCGAACCG
Rmar_0925	*Rm*Nag3	104.5	Forward	5′-CGAATTCCATATGCGCTCCGAACTCGATACCG
MG457246			Reverse	5′-CGCAGATCTCGGCAAGAGCGCGTTCA
Rmar_1080	*Rm*Xyl3A	87.2	Forward	5′-CCAATTGCATATGGAGCGACCGGCGTATCTGGAC
MG457247			Reverse	5′-CGCAGATCTTCGTCCCAGGTTACAGCCCTC
Rmar_1081	*Rm*Xyl3B	88.2	Forward	5′-CCAATTGCATATGGAAGTGCGCGTCGAGGACCTG
MG457248			Reverse	5′-CGGAGATCTGGGCCCCAGATTGCAGTCTTC
Rmar_2069	*Rm*Bgl3B	90.4	Forward	5′-CCAATTGCATATGTCCCGCGTGGATTCGTT
MG457249			Reverse	5′-CCCAAGCTTCTACTGCACGTCGATTTCCGTCTG
Rmar_2616	*Rm*Bgl3C	65.3	Forward	5′-CCAATTGCATATGGAACAGCAACCCCCTTCGAC
MG457250			Reverse	5′-CGGAGATCTGTAGGTCAGGCCATAGCCGAAG

[a] Restriction sites for *NdeI, BglII* and *HindIII* are underlined.

Supplementary Table 3. Hydrolytic activity of GH3 enzymes from *Rhodothermus marinus* DSM 4253 on *p*-nitrophenyl-glycosides.

Substrate	Relative activity[a] (%)					
	*Rm*Bgl3A *Rmar_0536*	*Rm*Bgl3B *Rmar_2069*	*Rm*Bgl3C *Rmar_2616*	*Rm*Xyl3A *Rmar_1080*	*Rm*Xyl3B *Rmar_1081*	*Rm*Nag3 *Rmar_0925*
*p*NP-β-Glc	100	92	46	5	10	–[b]
*p*NP-β-Cellobiose	100	63	–	–	–	–
*p*NP-β-Xyl	20	43	–	66	100	–
*p*NP-α-L-Ara	–	61	–	43	100	–
*p*NP-β-Glc*N*Ac	–	–	–	–	–	100
*p*NP-β-Gal	–	–	–	–	–	–
*p*NP-β-Man	–	–	–	–	–	–

[a] The highest specific activity was expressed as 100 % and the other specific activity was made relative to that.
[b] No activity was detected.

Supplementary Table 4. pH and temperature activity profile of GH3 enzymes from *Rhodothermus marinus* DSM 4253.

Enzyme	Optimum pH	Optimum temperature (°C)
*Rm*Bgl3A	5.4 ± 0.4	80
*Rm*Nag3	5.4 ± 0.4	90
*Rm*Xyl3A	5.6 ± 0.6	60
*Rm*Xyl3B	5.6 ± 0.6	60
*Rm*Bgl3B	5.4 ± 0.7	90
*Rm*Bgl3C	7.0 ± 0.1	70
